# Enhanced elastic stability of a topologically disordered crystalline metal–organic framework

**DOI:** 10.1038/s41563-024-01960-7

**Published:** 2024-07-23

**Authors:** Emily G. Meekel, Phillippa Partridge, Robert A. I. Paraoan, Joshua J. B. Levinsky, Ben Slater, Claire L. Hobday, Andrew L. Goodwin

**Affiliations:** 1 Inorganic Chemistry Laboratory, Department of Chemistry, University of Oxford, Oxford, UK; 2 Centre for Science at Extreme Conditions and EaStCHEM School of Chemistry, University of Edinburgh, Edinburgh, UK; 3 Department of Chemistry, University College London, London, UK

**Keywords:** Mechanical properties, Mechanical properties, Metal-organic frameworks

## Abstract

By virtue of their open network structures and low densities, metal–organic frameworks (MOFs) are soft materials that exhibit elastic instabilities at low applied stresses. The conventional strategy for improving elastic stability is to increase the connectivity of the underlying MOF network, which necessarily increases the material density and reduces the porosity. Here we demonstrate an alternative paradigm, whereby elastic stability is enhanced in a MOF with an aperiodic network topology. We use a combination of variable-pressure single-crystal X-ray diffraction measurements and coarse-grained lattice-dynamical calculations to interrogate the high-pressure behaviour of the topologically aperiodic system TRUMOF-1, which we compare against that of its ordered congener MOF-5. We show that the topology of the former quenches the elastic instability responsible for pressure-induced framework collapse in the latter, much as irregularity in the shapes and sizes of stones acts to prevent cooperative mechanical failure in drystone walls. Our results establish aperiodicity as a counter-intuitive design motif in engineering the mechanical properties of framework structures that is relevant to MOFs and larger-scale architectures alike.

## Abstract (web summary)

High-pressure experiments performed on aperiodic TRUMOF-1 demonstrate that this material remains crystalline up to pressures of 1.8 GPa, higher than other cubic metal–organic framework, due to the heterogeneous distribution of different shock-absorption mechanisms throughout the material.

## Main

Metal–organic frameworks (MOFs) are porous crystalline materials, the scaffolding-like structures of which are assembled from inorganic nodes connected by organic linkers^1^. Varying the geometry and connectivity of node and linker components allows access to an enormous variety of different network topologies^2^. From a mechanical perspective, MOFs are substantially more elastically compliant than conventional inorganic solids^3–5^, with moduli approaching those typical of organic polymers such as rubber^4^. This mechanical softness is primarily a consequence of the open network architecture of MOFs and the flexibility of their structural elements^6^; it is also a key ingredient in driving anomalous mechanical phenomena such as negative linear compressibility^7^ and negative gas adsorption^8^. However, the low elastic moduli of their structures mean that, when evacuated, MOFs are readily susceptible to collapse under applied pressure, such that they undergo pressure-induced amorphization or phase transitions to dense polymorphs^9^. Such low-pressure collapse is an important consideration for practical applications, as many industrial processes involve stresses greater than the typical elastic stability limits of MOFs^10^. The critical pressure at which deformation occurs—itself closely related to the magnitude of the elastic moduli^11^—scales loosely with network connectivity and the strength of the metal–linker interactions^12,13^. For example, the four-connected zeolitic imidazolate framework ZIF-8 (Zn^2+^–N links) amorphizes at 0.34 GPa whereas the 12-connected framework UiO-66 (Zr^4+^–O links) maintains crystallinity up to a threshold of 1.4 GPa (refs. ^10,14,15^). The corresponding shear moduli are 0.97 and 14 GPa, respectively^12,16^. Hence, the conventional design focus for strengthening MOFs has centred primarily on high-connectivity network structures assembled from highly charged cations^17^.

In entirely different contexts, it is well appreciated that irregular architectures can confer mechanical strength by frustrating collapse mechanisms^18–21^. An archetypal example is using various sizes and shapes of stone to strengthen ‘drystone wall’ structures. Another is the complex disordered channel networks in termite nests, which optimize structural stability for given resources and which inspired a new class of advanced metamaterials^20,22,23^. An obvious question is whether these same ideas might be relevant on the atomic scale to improve the elastic stability of functional materials such as MOFs.

**Fig. 1:**
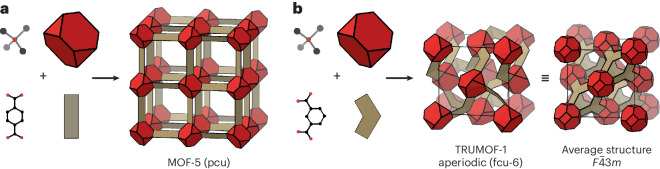
Assembly of MOF-5 and TRUMOF-1 structures from building units. **a**, In MOF-5, tetrahedral OZn_4_ clusters (red polyhedra) are connected by 1,4-bdc linkers (beige panels) to form a network structure with the simple cubic pcu topology. **b**, The structure of TRUMOF-1 is generated by similar components, except that the linear 1,4-bdc linker is replaced by the bent 1,3-bdc isomer (beige angled panels). Clusters are arranged on a face-centred cubic lattice, with each cluster connected to six of its 12 nearest neighbours (we call this topology fcu-6). The connectivity in TRUMOF-1 is aperiodic. Shown in the centre of this panel is a representation of one possible 1 × 1 × 1 approximant. The configurational average of the TRUMOF-1 structure, which is periodic but with partial site occupancies, is represented on the right-hand side.

We have recently discovered the material TRUMOF-1, a crystalline MOF whose structure is based on an unusual aperiodic network topology related to so-called Truchet tilings^24,25^. The chemistry of TRUMOF-1 is almost identical to that of the canonical system MOF-5. Both are assembled from octahedrally coordinated OZn_4_ clusters connected by benzenedicarboxylate (bdc) linkers to form a network of uniformly six-connected nodes (Fig. 1a,b)^1^. For MOF-5, the linear connectivity of 1,4-bdc links nodes to give a network with the simple cubic (pcu) topology. The nodes of TRUMOF-1 are also six-connected with local octahedral geometries, but the network formed by the bent 1,3-bdc linkers is aperiodic and never repeats^24^. The resulting connectivity is based on non-random partial occupancy of the 12-connected fcu net in a way that is locally homogeneous but not long-range ordered. In the absence of a formal language for such aperiodic networks, we use the symbol fcu-6 to denote this aperiodic six-connected variant of the fcu topology. The key point is that the two materials share the same degree of network connectivity and the same node–linker chemistry but by virtue of a subtle change in linker geometry, they differ in terms of the presence or absence of topological periodicity.

Here we report the use of variable-pressure single-crystal X-ray diffraction measurements to study the elastic behaviour of TRUMOF-1 under hydrostatic compression^26^. Whereas evacuated MOF-5 is famously unstable under pressure (it amorphizes at *p* ≲ 0.2 GPa; refs. ^27–29^), we found that TRUMOF-1 remains crystalline to the hydrostatic limit (1.8 GPa) of a suitable non-penetrating pressure-transmitting medium. Indeed, our experimental measurements establish TRUMOF-1 as the most elastically stable, isotropic MOF reported to date. This context of isotropy is conceptually important because anisotropic MOFs have access to additional strain responses that facilitate volume reduction without symmetry breaking (for example, wine-rack flexing). Using a coarse-grained lattice-dynamical model that captures the key mechanical effects of topological aperiodicity in TRUMOF-1, we rationalize the enhancement of elastic stability of this system relative to MOF-5 and demonstrate that its compression mechanism involves the activation of internal degrees of freedom in a way that varies spatially throughout the TRUMOF-1 structure. We argue that these displacements provide a shock-absorption mechanism that is closely related to both the combinatorial mechanics proposed in ref. ^30^ and the spatially inhomogeneous mechanical responses identified in disordered metamaterials^20,31^. Our study suggests how aperiodic network architectures of the kind adopted by TRUMOF-1 might be exploited in the rational design of low-density framework structures with useful mechanical properties.

## High-pressure crystallography

**Fig. 2:**
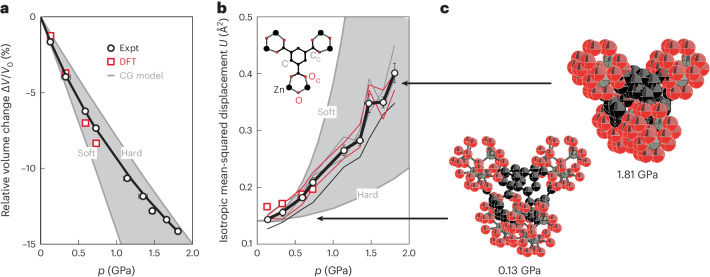
Structural response of TRUMOF-1 to hydrostatic pressure. **a**, The *V*(*p*) equation of state obtained using single-crystal X-ray diffraction measurements (open circles) is well accounted for by a third-order Birch–Murnaghan fit (solid line). The errors for *p* and *V* are given for the uncertainties in the measurement as described in Methods. The low-pressure behaviour obtained from DFT-driven geometry optimizations of a 2 × 2 × 2 approximant is shown as open squares and that of our coarse-grained lattice-dynamical models (CG model) is shown as a shaded region. **b**, Isotropic mean-squared displacement magnitudes extracted from our crystallographic analysis increase with pressure. Trends for individual atom types are shown as thin lines, with the average ± standard error of the mean shown as open circles with error bars and a bold line. The errors for *p* are given for the uncertainties in the measurement as described in Methods. Estimated values extracted from our DFT configurations are shown as open squares. The CG and DFT data have been shifted vertically by 0.14 Å^2^ as an estimate for the thermal contribution to *U* that most closely aligns experiment and calculation. **c**, Representations of fragments of the TRUMOF-1 structure at low and high pressure, with displacement ellipsoids shown at 50% probability. Zn, O and C atoms are shown in grey, red and black, respectively. Error bars represent standard errors obtained from our crystallographic refinements. Expt, experimental. Source data

The variable-pressure X-ray diffraction behaviour of evacuated TRUMOF-1 single crystals is relatively straightforward. Throughout the hydrostatic regime of the non-penetrating pressure-transmitting medium used in our study (Daphne oil 7373; 0–1.8 GPa)^32^, we observed that pressure effected a smooth and monotonic decrease in the size of the cubic *F*$\bar{4}$3*m* unit cell of TRUMOF-1 (Fig. 2a). In particular, there was no evidence for any symmetry-lowering phase transitions from the ambient-pressure phase nor was there any indication of pressure-induced amorphization. As straightforward as this behaviour may seem, it is, nonetheless, surprising. Our results show that TRUMOF-1 outperforms all known isotropic MOFs in terms of stability under hydrostatic compression^5,26^. This resilience to applied pressure is not a consequence of TRUMOF-1 being particularly stiff. The experimental *V*(*p*) equation of state corresponds to a zero-pressure bulk modulus *B*_0_ = 7.5(5) GPa, which is much lower than that of MOF-5 (15–40 GPa)^13,29,33^. The pressure derivative of the bulk modulus, determined using a third-order Birch–Murnaghan fit to our data, is *B*′ = 5.9(7). Density functional theory (DFT) calculations carried out for a set of ten 1 × 1 × 1 approximants^24^ of the TRUMOF-1 structure gave values of the zero-pressure bulk modulus across the range 4.8 ≤ *B*_0_ ≤ 7.4 GPa, the larger values correlating with denser approximant structures. Geometric optimization of a larger set of approximants (2 × 2 × 2) for a series of low applied pressures gave volume reductions that were close to our experimental values (Fig. 2a; see also Methods, Supplementary Note 1, Supplementary Fig. 1 and Supplementary Table 1). Mindful of the complexity of TRUMOF-1, we consider this agreement between DFT and experiment to be remarkably good. So, our first key finding is that TRUMOF-1 is a compliant material that is, nonetheless, able to accommodate compression to a much larger degree than other, stiffer MOFs.

Our diffraction data were sufficiently complete to allow crystal-structure refinement as a function of applied pressure. These refinements considered only the Bragg component to the diffraction pattern (the diffuse scattering interpreted in ref. ^24^ being too weak for us to measure accurately in a pressure cell). We found no meaningful change to the average structure of TRUMOF-1, other than a strong increase in the magnitude of atomic displacement parameters with increasing pressure (Fig. 2b,c). An analysis of the residual electron density in Fourier maps showed no appreciable variation in pore content with pressure (Supplementary Note 2 and Supplementary Table 2), which is consistent with the exclusion of the pressure-transmitting medium from the pore network throughout our measurements^29,32^. Hence, our results can be interpreted in terms of the intrinsic behaviour of TRUMOF-1 itself and free from guest-inclusion effects^9,29,34^.

The increase in magnitude of atomic displacements with pressure is at once both anomalous and counter-intuitive. After all, densification usually dampens vibrational motion such that any measure of atomic displacements decreases accordingly as pressure is increased^35^. This effect is captured formally by the Grüneisen parameter $\gamma =-\partial \ln\omega /\partial \ln{V}$, which links changes in volume *V* to changes in phonon frequencies *ω* (ref. ^36^). In most materials, $\gamma \simeq 1$, and hence phonons stiffen under pressure, which reduces the amplitude of thermal motion. Exceptions are known, and perhaps the best studied are those of phonon-driven negative thermal expansion materials such as ScF_3_ (ref. ^37^), for which *γ* < 0 and the amplitude of volume-reducing phonon modes actually increases as the volume is reduced^38^. TRUMOF-1, however, does not show negative thermal expansion^24^, and we will come to rationalize the anomalous increase in displacement magnitude in terms of an alternative mechanism whereby pressure magnifies the degree of static disorder.

## Coarse-grained elastic modelling

At face value, there is no obvious reason why TRUMOF-1 should be so much more elastically stable than MOF-5. After all, the two systems share the same chemistry and the same degree of network connectivity. To understand how their topological differences result in such contrasting mechanical responses, we sought to develop the simplest possible lattice-dynamical model capable of capturing the key elastic properties of the two systems. Our approach (inspired by that used elsewhere^39^ for ScF_3_) was as follows. First, we developed a simplified model of MOF-5 in which OZn_4_ clusters were mapped onto individual sites and linkers were mapped onto harmonic springs connecting one site to its six nearest neighbours. Further harmonic (three-body) springs were included among neighbouring triplets to capture the angular rigidity of the network. In this way, the lattice energy of MOF-5 is coarse-grained as follows:

1 $${E}_{{\rm{latt}}}=\frac{1}{2}{k}_{r}\sum _{i > j}{({r}_{{ij}}-{r}_{{\rm{e}}})}^{2}+\frac{1}{2}{k}_{\theta }\sum _{i > j > k}{({\theta }_{{ijk}}-{\theta }_{{\rm{e}}})}^{2},$$

where *k*_*r*_ and *k*_*θ*_ are effective force constants that capture the resistance of MOF-5 to linear and shear deformations, respectively. The sums in equation (1) are taken over connected nodes *i* and *j* (and *k*), *r*_*ij*_ and *θ*_*ijk*_ are the inter-node distances and angles, and *r*_e_ and *θ*_e_ represent the equilibrium node separation (half the MOF-5 unit-cell parameter) and the relevant intra-framework angle (90° or 180° as appropriate). The two free parameters of this model—namely the numerical values of *k*_*r*_ and *k*_*θ*_—were fixed so as to reproduce the *C*_11_ and *C*_44_ elastic constants of MOF-5. As it happens, there is a large spread among measures of these values reported from both experimental and computational studies^13,40,41^, so we consider limiting cases of both ‘soft’ and ‘hard’ parameterizations (Methods).

**Fig. 3:**
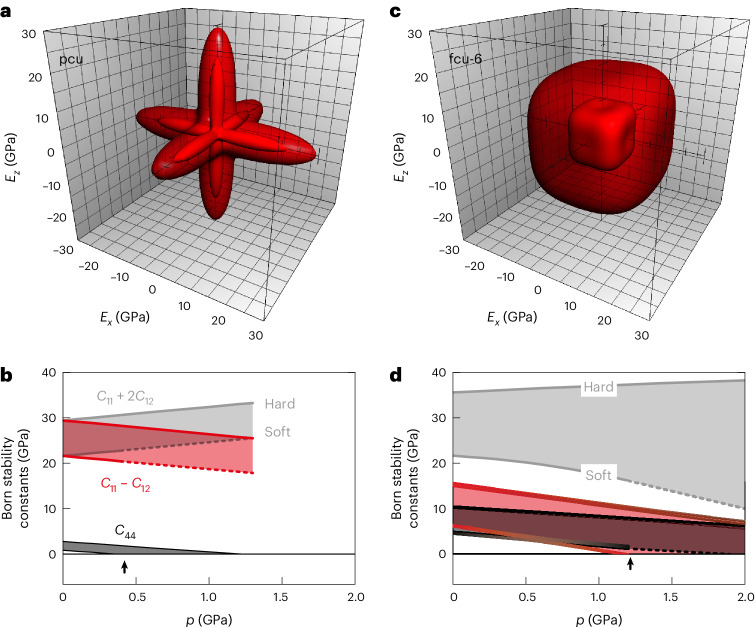
Elastic behaviour of the coarse-grained MOF-5 and TRUMOF-1 models. **a**, Orientational dependence of the Young’s modulus calculated from equation (1) applied to the pcu topology of MOF-5, parameterized as discussed in the text. The soft (hard) parameter set gives rise to the interior (exterior) surface of the indicatrix as drawn. **b**, Pressure dependence of the Born stability constants for this pcu model, defined as the eigenvalues of the elastic stiffness tensor *B* under load^11,55^. Shaded regions correspond to the values spanned by the soft and hard parameterizations discussed in the text. The onset of elastic instability is marked by an arrow. **c**, The orientational Young’s modulus surfaces for the coarse-grained TRUMOF-1 structure with the fcu-6 topology, shown using the same representation used in **a** for MOF-5. **d**, Pressure dependence of the Born stability constants for our coarse-grained TRUMOF-1 model, which reveals an enhanced elastic stability relative to MOF-5. Formally, the absence of crystallographic symmetry in the approximant structure allows arbitrary mixing of the eigenstates of the elastic tensor. We have coloured the stability constants by similarity to the *C*_11_ + 2*C*_12_, *C*_11_ − *C*_12_ and *C*_44_ eigenstates shown in **b**. Dashed lines in **b** and **d** denote extrapolations beyond the corresponding elastic stability regime. Source data

Irrespective of the particular parameterization employed, the elastic behaviour described by equation (1) captures qualitatively the key response of MOF-5 to external stress. For example, the orientational dependence of the Young’s modulus^42^ is like that identified in earlier studies, in terms of both degree and form of anisotropy (Fig. 3a) (see Supplementary Note 3 and Supplementary Fig. 2 (refs. ^4,13,33^)). Likewise, as the pressure is increased, the *C*_44_ elastic constant softens, with the system becoming mechanically unstable beyond *p* ≃ 0.3 GPa (Fig. 3b). As the connectivity in equation (1) is fixed, this simple model cannot reproduce amorphization; however, the shear instability of MOF-5 is considered a key element in driving amorphization in practice, and it also places an upper bound on the pressure stability of the material^11,43^.

Our next step was to transpose this coarse-grained model to TRUMOF-1, while retaining the same range of effective spring constants *k*_*r*_ and *k*_*θ*_ used for MOF-5 (as the chemistries are so similar) and using the aperiodic fcu-6 network connectivity identified in ref. ^24^ to assign neighbour pairs and triplets among which these harmonic interactions operate. The equilibrium value *r*_e_ was set to the node–node separation of the TRUMOF-1 structure (*a*/√2), and the equilibrium angles *θ*_e_—now a function of triplet *i*, *j* and *k*—were set to the corresponding values when nodes *i*, *j* and *k* were placed at the high-symmetry (face-centred) sites in the configurationally averaged *F*$\bar{4}$3*m* TRUMOF-1 cell. In this way, the ground state of equation (1), when applied to TRUMOF-1, corresponds to a face-centred cubic arrangement of nodes, as observed in the experimental average structure. Our use of a coarse-grained model allows interrogation of much larger approximants to the aperiodic structure of TRUMOF-1 than are accessible using, for example, DFT.

The elastic behaviour of this simplified TRUMOF-1 model turns out to be interesting in a number of respects. First, the Young’s modulus is now very isotropic (Fig. 3c and Supplementary Fig. 3). Using the metric of ref. ^42^, we obtained an elastic anisotropy 1.3 < *E*_max_/*E*_min_ < 1.5 much reduced from that of MOF-5 (4.0 < *E*_max_/*E*_min_ < 9.3). Indeed, the form of the Young’s modulus that we obtained qualitatively resembles those of the two isotropic periodic elastic networks (crs and hxg) derivable from the fcu topology by removing half of the elastic links (Supplementary Note 4 and Supplementary Fig. 4). Second, the system remains elastically stable well beyond 1 GPa (Fig. 3d). In other words, the enhanced elastic stability observed experimentally is successfully reproduced by this coarse-grained model. Moreover, the absence of any crystallographic symmetry ensures that the system is dynamically stable (with real-valued phonon frequencies) throughout this same pressure range^44^. Third, the *V*(*p*) equation of state gives a low-pressure bulk modulus 7.2 < *B*_0_ < 12 GPa that is remarkably close to the experimental value (Fig. 1a). The only qualitative feature of *V*(*p*) not captured by the coarse-grained model is the curvature at high pressure, which is perhaps unsurprising given the omission of any non-bonded interactions. These various results are essentially independent of the specific approximant network used, with larger approximants exhibiting smaller variances and greater elastic stabilities (Supplementary Note 5 and Supplementary Fig. 5). Consequently, we conclude that the contrasting elastic stabilities of MOF-5 and TRUMOF-1 can, indeed, be rationalized in terms of the difference in the topologies of their underlying elastic networks.

## Spatially heterogeneous mechanical response

**Fig. 4:**
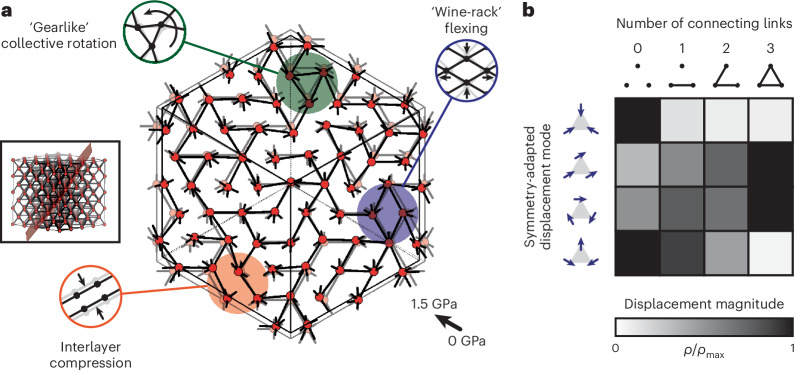
Heterogeneous compression mechanism of TRUMOF-1. **a**, Representation of characteristic node displacements induced by compression of TRUMOF-1, shown here for a (111) slice of a 4 × 4 × 4 approximant (inset). Compression mechanisms vary spatially, and some easily interpreted mechanical motifs are highlighted by coloured circles. **b**, The displacement patterns of neighbouring triplets of nodes can be decomposed by symmetry into, from top to bottom, breathing, translational, rotational and bending modes. The relative population *ρ*/*ρ*_max_ for each mode varies as a function of the local elastic connectivity. The values given here are averages over the mode displacements activated on compression to 1 GPa among ten 4 × 4 × 4 approximants. Source data

Whereas in MOF-5 the relative arrangements of nodes within the unit cell are unaffected by pressure (their positions being fixed by crystal symmetry), the equilibrium node positions in TRUMOF-1 are free to vary as pressure is applied. The spatially varying network connectivity then results in an increased displacement of nodes away from their high-symmetry sites at elevated pressures (Fig. 4a). One would expect to see a signature of this behaviour in the crystallographic model, which represents a configurational average over all unit cells. We extracted from our lattice-dynamical calculations the pressure dependence of the mean-squared node displacements *U*(*p*), which is a measure of the additional static disorder introduced by spatial variations in the elastic network of TRUMOF-1. This function maps closely onto the increase in magnitude of atomic displacements measured experimentally (Fig. 2b). Our DFT configurations, which necessarily involve smaller unit cells with higher crystal symmetry, respond to pressure through a combination of internal reorganization and increasing anisotropic strain. We found that the latter dominates, but this contribution can, nonetheless, be recast in terms of a static disorder contribution, which again follows the experiment relatively closely (Fig. 2b and Methods). Hence, we attribute the anomalous increase in the value of *U* with pressure to an increase in the degree of static disorder of the underlying TRUMOF-1 network, itself a consequence of topological aperiodicity. For context, note that a related effect has been observed previously in the low-temperature crystallography of various MOFs, for which spatial inhomogeneities in host–guest interactions induced by cooling result in an anomalous increase in *U* with decreasing *T* (refs. ^45,46^).

The node displacement patterns that emerge from our simple lattice-dynamical calculations provide further qualitative insight into the pressure-induced mechanical response of TRUMOF-1. Macroscopic strain is accommodated through large-scale node displacements that, although correlated between connected nodes, are, nonetheless, spatially localized and do not propagate cooperatively. This localization may act to frustrate collective instabilities in a manner analogous to that proposed elsewhere for stiffening disordered metamaterials^20^. An interesting feature of the distortion pattern that emerges, as shown in Fig. 4a, is that different mechanisms (for example, wine-rack flexing^47^, layer compression^48^ and even gearlike motion^49^) are activated in different regions of the aperiodic network (Supplementary Video 1). That disordered networks might provide a material with a library of distinct elastic response mechanisms that can be amplified selectively so as to best accommodate a given strain is a concept termed ‘combinatorial mechanics’, which is the mechanical analogue of the response of dynamic combinatorial libraries to chemical perturbation^30,50^. Whereas in randomly diluted elastic networks bulk elasticity is dominated by the existence (or absence) of a single percolating cluster^51^, the uniform local connectivity in TRUMOF-1 appears to drive a more complex and spatially distributed response. We note in passing a conceptual similarity to strain engineering of UiO-66 through the incorporation of linker vacancies, so that again counter-intuitive mechanical responses emerge from the interplay of correlated defects^52^.

To place our analysis on a semi-quantitative standing, we have included in Fig. 4b a summary of the relative populations of some different kinds of local distortions due to compression. For ease of interpretation, we focus on the displacements of node triplets under pressure. After all, these can be visualized easily in the two-dimensional slice representation we use here. However, the same analysis might, in principle, be extended to three dimensions by interpreting the displacements of nodes surrounding, for example, the tetrahedral or octahedral holes of the underlying face-centred cubic lattice. The four kinds of symmetry-adapted displacement patterns for node triplets (breathing, translation, rotation and bending) are illustrated graphically in Fig. 4b. There is a strong correlation between the relative population of a given pattern and the elastic connectivity of the three nodes in the triplet. These correlations are entirely intuitive and recur among different approximants and for different absolute pressures. Node triplets that are elastically unconnected tend to distort, and as the number of connections is increased the triplet tends to displace as a collective unit. Further analysis and discussion on this point are given in Supplementary Note 6, Supplementary Fig. 6 and Supplementary Table 3.

## Outlook

Given the strong coupling between local elastic connectivity and the corresponding mechanical response of the fcu-6 network, one would expect fundamental differences in the vibrational properties of TRUMOF-1 relative to those of conventional periodic network structures. In particular, the spatial localization of different response mechanisms suggests a breakdown of the usual phonon description for this material, with mode broadening in both wavevector and energy^53^. Such behaviour is known from theory to arise in related systems based on strongly correlated disorder^54^, where the interest is in exploiting anomalous phonon broadening as a design principle for inhibiting thermal transport in thermoelectrics. Hence, the anomalous mechanical response of TRUMOF-1 to hydrostatic pressure (as we report here) may also provide insight in due course into the dynamical response of the same material as a function of temperature. We anticipate that Truchet-tile architectures may allow the engineering of anomalous thermal responses in precisely the same way that we have now found that TRUMOF-1 exhibits a combination of elastic compliance and elastic stability not observed in conventional, crystalline MOFs.

## Methods

### Synthesis

Single crystals of TRUMOF-1 were synthesized by combining solutions of Zn(NO_3_)_2_·6H_2_O (0.33 mmol) and 1,3-benzenedicarboxylic acid (0.33 mmol) in *N*,*N*-dimethylformamide (5 ml). The resulting mixture was placed in a Teflon-lined solvothermal vial, sealed and then heated to 110 °C in an oven. The temperature was maintained at this value for 14 h, after which time the system was slowly cooled to room temperature. TRUMOF-1 crystals, which formed as colourless truncated pyramids, were isolated by filtration and washed three times with chloroform. Each washing cycle involved soaking TRUMOF-1 crystals in fresh CHCl_3_ for 24 h, which was followed by filtration.

### Variable-pressure crystallography

A single crystal of TRUMOF-1 was soaked in CHCl_3_ and evacuated under vacuum before being loaded into a Merrill–Bassett diamond anvil cell with a half-opening angle of 40°. The latter was composed of Boehler-Almax diamonds with 600 µm culet diamond anvils, a tungsten gasket and tungsten carbide backing plates^56^. A small ruby chip was also loaded into the cell to act as an internal pressure calibrant. The pressure-dependent fluorescence of the ruby was used to measure the pressure^57^. Daphne oil 7373 (ref. ^32^) was added as a pressure-transmitting medium. High-pressure X-ray diffraction experiments were performed at the I19 beamline at the Diamond Light Source over a pressure range 0.13 ≤ *p* ≤ 1.81 GPa (specifically at pressures 0.13, 0.33, 0.59, 0.73, 1.14, 1.35, 1.47, 1.66 and 1.81 GPa). Data collection involved a step size, transmission and exposure time of 1, 3% and 0.3 s, respectively. Unit-cell determination, data integration, frame scaling and absorption corrections were performed using CrysAlis^Pro^ (ref. ^58^).

Structure refinements were carried out for the dataset collected at 0.13 GPa, as outlined in ref. ^24^, albeit using a simplified disorder model in which the split carbon sites C2A and C2B were combined into a single ‘C2’ site with occupancy 0.75. This crystallographic model of TRUMOF-1 was then used in the refinement of all other high-pressure datasets. To maintain consistency over all datasets and account for the data limitations arising from the experimental set-up, the atomic displacement parameters for all atoms were refined isotropically. The FLAT, DFIX and SADI constraints were used on the C2 and C3 atoms to fix the geometry of the benzene ring. The H atoms were placed geometrically and constrained to ride on their host atoms. Because the C2A and C2B sites were not distinguished, the H2B site of ref. ^24^ was not included in this model. This accounts for the reduced hydrogen content in the crystallographic model (empirical formula C_24_H_9_O_13_Zn_4_) relative to the true empirical formula C_24_H_12_O_13_Zn_4_. The asymmetric units of all datasets are overlaid in Supplementary Fig. 7, with the opacity decreasing with pressure. The relevant crystallographic tables are Supplementary Tables 4–12. The corresponding CIFs and checkCIFs are provided as Supplementary Data 1.

### Equation of state

A third-order Birch–Murnaghan fit for the equation of state of TRUMOF-1 was performed using the web tool Pascalapp (ref. ^59^). The resulting values for the bulk modulus *B*_0_ and its derivative *B*′ are 7.5(4) GPa and 5.9(7), respectively. The expected pressures from the third-order fit using these parameters are given alongside their corresponding unit-cell volumes and experimental pressure values in Supplementary Table 13.

### Coarse-grained lattice-dynamical modelling

To capture the role of topology and aperiodicity in the differing mechanical behaviours of MOF-5 and TRUMOF-1, a simple model was built in which [OZn_4_] clusters were represented by atoms and the bdc linker connections between these were replaced by pseudo-springs. Angular harmonic interactions between connected triplets of atoms were also included to model resistance to bond angle deformations. These models were written as GULP input files^60^ denoted by their respective topologies: MOF-5 as pcu and TRUMOF-1 as aperiodic fcu-6 (Supplementary Data 2). The pcu input corresponded to a single primitive cubic unit cell, whereas fcu-6 was represented as a 4 × 4 × 4 approximant supercell, both with periodic boundary conditions. The connectivity within the fcu-6 supercell was generated using the Monte Carlo algorithm described in ref. ^24^, which ensured that every node was connected to exactly six others in a manner that respected the geometric constraints of TRUMOF-1. The resulting coarse-grained lattice energy was given by equation (1).

The equilibrium bond lengths for each model *r*_e_ were chosen to coincide with the crystallographically determined separations of clusters in MOF-5 and TRUMOF-1, 12.946 and 10.898 Å, respectively. Likewise the bond angles were taken as 90° and 180° for pcu and 60°, 90°, 120° and 180° as appropriate in fcu-6. The harmonic spring constants *k*_*r*_ and *k*_*θ*_ were chosen to reproduce the *C*_11_ and *C*_44_ elastic moduli of MOF-5 as determined previously by DFT methods. As a range of values are reported, lower and upper range values were chosen for each to give the two limiting parameterizations referred to as ‘hard’ and ‘soft’ in Main. The hard and soft values were *k*_*r*_ = 2.376 eV Å^−2^ and *k*_*θ*_ = 9.15 eV rad^−2^ (to match *C*_11_ = 29.4 GPa (ref. ^40^) and *C*_44_ = 2.7 GPa (ref. ^41^)) and *k*_*r*_ = 1.7455 eV Å^−2^ and *k*_*θ*_ = 2.72 eV rad^−2^ (to match *C*_11_ = 21.6 GPa and *C*_44_ = 0.8 GPa (ref. ^13^)), respectively.

The GULP output for the fcu-6 calculations included the elastic tensor *C*, the approximant cell lattice parameters and the equilibrium node coordinates at a given pressure. The value of *C* was input to the ELATE code, which was used to determine the orientational dependence of the Young’s moduli^42^. The pressure dependence of the lattice parameters gave the equation of state shown in Fig. 2. The equilibrium node coordinates were used to estimate the degree of pressure-dependent static disorder, as discussed in ‘Estimating the pressure-induced static disorder’.

### Born stability criteria

The elasticity matrix *C*, obtained from the GULP calculations, was adjusted to form the pressure-dependent second-order stiffness tensor:

2 $${{B}}={{C}}-p\begin{bmatrix}-1&1&1&0&0&0\\1&-1&1&0&0&0\\ 1&1&-1&0&0&0\\ 0&0&0&-1&0&0\\ 0&0&0&0&-1&0\\ 0&0&0&0&0&-1\end{bmatrix}.$$

The Born stability criteria were considered violated when at least one of the eigenvalues of *B* was negative^61^.

### Estimating the pressure-induced static disorder

The pressure dependence of the equilibrium node coordinates obtained using GULP was used to estimate the increase of static disorder under pressure. Node displacements were first corrected for origin shift. The supercell was then projected onto its parent (1 × 1 × 1) cell, face-centring operations applied and the mean-squared displacement calculated.

### Symmetry-adapted node displacements

For a given 4 × 4 × 4 approximant, we compared the relative coordinates of each node *i* at 0 and 1 GPa to determine a set of displacement vectors **u**_*i*_. We corrected each vector for configurational drift by subtracting the average displacement: **u**_*i*_′ = **u**_*i*_ − 〈**u**〉. For each triplet of neighbouring nodes, we determined a suitable rotation matrix *R*_*ijk*_ that transformed the set of nodes *i*, *j* and *k* onto a common orientation and then projected the node displacement vectors onto the plane containing the nodes in the 0 GPa structure. The set of node displacement vectors (each now two dimensional) were then assembled into a six-component displacement vector **e**_*ijk*_. The symmetry-adapted displacement vector

3 $${\widetilde{{\bf{e}}}}_{{ijk}}=\left[\begin{array}{cccccc}0 & 1/\surd 3 & 1/2 & -1/\surd 12 & -1/2 & -1/\surd 12\\ 1/\surd 3 & 0 & 1/\surd 3 & 0 & 1/\surd 3 & 0\\ 0 & 1/\surd 3 & 0 & 1/\surd 3 & 0 & 1/\surd 3\\ 1/\surd 3 & 0 & -1/\surd 12 & -1/2 & -1/\surd 12 & 1/2\\ 0 & 1/\surd 3 & -1/2 & -1/\surd 12 & 1/2 & -1/\surd 12\\ 1/\surd 3 & 0 & -1/\surd 12 & 1/2 & -1/\surd 12 & -1/2\end{array}\right]{{\bf{e}}}_{{ijk}},$$

then contains as its elements the displacements for each normal mode. The transformation matrix used here gives, in order, the breathing (*A*_1_′), translational (*E*′), rotational (*A*_2_′) and bending (*E*′) mode amplitudes. We have used the irreducible representation labels of the *D*_3*h*_ point group appropriate for a triangle of neighbouring nodes. The average mode amplitudes *ρ*_*α*_ = 〈(4*a*${\widetilde{e}}_{\alpha }$)^2^〉 (where *a* is the TRUMOF-1 unit-cell constant) and their estimated standard errors (determined from ten 4 × 4 × 4 approximants) are listed in Supplementary Table 3, together with the relative values *ρ*/*ρ*_max_ given in Fig. 4b.

### DFT calculations

All calculations were performed using CP2K v.2023.2 (www.cp2k.org). Three sample 2 × 2 × 2 TRUMOF-1 configurations were selected from our previous study^24^. These corresponded to the least stable and least dense structure (labelled V), the densest and most stable structure (XIII) and a structure with intermediate density and intermediate stability (II). All structures contained 1,696 atoms. All configurations were fully relaxed to the equilibrium density. All cell parameters were independently optimized at 0, 0.13, 0.33, 0.59 and 0.73 GPa. The cutoff used was 950 Ry with a relative cutoff of 60 Ry and the TZV2P basis reported by the University of Zurich group (available with the CP2K data download). Dispersion corrections were applied using the Grimme D3 approach^62^ and the Perdew–Burke–Ernzerhof functional. The low-pressure pressure dependence of the relative volume change for the three approximant structures interrogated is shown in Supplementary Fig. 1. The behaviour of model XIII is that shown in Fig. 2. Corresponding cell parameters are listed in Supplementary Table 1, and converged DFT configurations are given as Supplementary Data 3.

The same CP2K basis set and cutoff settings were used in the calculations to estimate the bulk moduli for ten 1 × 1 × 1 cells as reported in ref. ^24^. After fully optimizing the cells to equilibrium density, the structures were then incrementally increased and decreased in 1% increments up to ±10% and re-optimized at the scaled volumes, giving a total of 21 structures that were used in the fitting of the Birch–Murnaghan equation of state.

The smaller size (2 × 2 × 2) of the DFT approximants relative to the 4 × 4 × 4 approximant used in our coarse-grained elastic representation of TRUMOF-1 corresponds to a greater effective crystal symmetry. One result of this increased symmetry is that the anisotropic compressibility of the approximant cell plays a larger role in accommodating deformation mechanisms in the DFT configurations than does internal reorganization of the node positions within the cell. To estimate the contribution of anisotropic cell distortions to the static disorder in node positions, we calculated the principal strain components ε_*i*_(*p*). The function

4 $$\Delta {U}_{\mathrm{iso}}(\;p)\simeq \frac{1}{3}\sum _{i}{\left(\frac{a{{\rm{\varepsilon }}}_{{i}}(\;p)}{2}\right)}^{2},$$

corrected for thermal motion, is plotted in Fig. 2b. Note that the essence of equation (4) is to quantify the degree of variance in node position introduced by orientational averaging over the anisotropic cells generated by DFT.

## Online content

Any methods, additional references, Nature Portfolio reporting summaries, source data, extended data, supplementary information, acknowledgements, peer review information; details of author contributions and competing interests; and statements of data and code availability are available at 10.1038/s41563-024-01960-7.

### Supplementary information

Supplementary Information Supplementary Notes 1–6, Figs. 1–7, Tables 1–13 and references.

Supplementary Data 1 Crystallographic data.

Supplementary Data 2 Representative GULP input file.

Supplementary Data 3 DFT configurations.

Supplementary Video 1 Animation of compression mechanism.

### Source data

Source Data Fig. 2 Equation of state data plotted in Fig. 2a and mean-squared displacement data plotted in Fig. 2b.

Source Data Fig. 3 Born stability constant data plotted in Fig. 3b,d.

Source Data Fig. 4 Displacement pattern data plotted in Fig. 4b.

## Data Availability

The authors declare that the data supporting the findings of this study are available within the paper and its Supplementary Information files. Source data are provided with this paper.
