# Supplementary material for: Enhanced elastic stability of a topologically disordered crystalline metal–organic framework
Source: Nat Mater. 2024 Jul 23;23(9):1245–51. doi: 10.1038/s41563-024-01960-7 (PMC11364505; doi:10.1038/s41563-024-01960-7)
Supplement: Supplementary file 1 — Supplementary Notes 1–6, Figs. 1–7, Tables 1–13 and references. [file 41563_2024_1960_MOESM1_ESM.pdf]

nature materials

Supplementary information https://doi.org/10.1038/s41563-024-01960-7

# Enhanced elastic stability of a topologically disordered crystalline metal–organic framework

In the format provided by the authors and unedited

## Contents

**Supplementary Notes** 2

**Supplementary Figures** 5

**Supplementary Tables** 10

**Supplementary References** 22

# Supplementary Notes

## Supplementary Note 1

To test the sensitivity of the predicted geometries to the choice of density functional, the most stable $1 \times 1 \times 1$ structure identified previously[24] with $P\bar{4}$ symmetry was expanded to a $2 \times 2 \times 2$ cell containing 1696 atoms and relaxed using the PBESOL functional,[63] a 950 Ry cutoff, a 80 Ry relative cutoff and the same TZV2P basis set and pseudopotentials as described above. A constant hydrostatic pressure was applied of 0, 0.13, 0.33, 0.59, 0.73 and 1.14 GPa, and the relative volume is displayed in Supplementary Figure 1. As for the results obtained for the $2 \times 2 \times 2$ approximants, the change in volume with pressure closely matches the experimental measurements, giving confidence that the approximants used capture the mechanical response mechanisms.

## Supplementary Note 2

The number of DMF solvent molecules per unit cell was calculated for each TRUMOF-1 high-pressure dataset using the solvent mask algorithm 'BYPASS' within OLEX2 (Supplementary Table 2).[64] Our key conclusion was that the residual electron density did not vary meaningfully at elevated pressure, and hence there is no evidence for inclusion of pressure-transmitting medium throughout the experiment. Note that at pressures higher than 1.14(5) GPa, refinement stability decreased as a consequence of the reduced diffraction intensities. Hence the calculated cavity electron density calculations are less accurate accurate at elevated pressures (threshold indicated by the horizontal line in Supplementary Table 2). In the specific case of the dataset collected at 1.47(4) GPa, the refinement was too unstable when the solvent mask algorithm was applied, and so no electron density values are reported for this case.

## Supplementary Note 3

The elastic stiffness matrices (in Voigt notation) for the **pcu** and **fcu-6** models with both parameterisation sets were obtained from GULP[60] at all pressure points for which the corresponding model was mechanically stable. The **fcu-6** GULP outputs were also used to qualitatively inspect its mechanisms of compression in CrystalMaker[65] when subject to isotropic loading. Supple-

mentary Video 1 is an animation of one of the (111) planes of the **fcu-6** supercell model during compression up to its instability point.

Representative elastic tensors at 0 GPa are as follows:

$$\mathbf{C}_{\text{pcu}} = \begin{pmatrix} 29.4 & 0 & 0 & 0 & 0 & 0 \\ 0 & 29.4 & 0 & 0 & 0 & 0 \\ 0 & 0 & 29.4 & 0 & 0 & 0 \\ 0 & 0 & 0 & 2.7 & 0 & 0 \\ 0 & 0 & 0 & 0 & 2.7 & 0 \\ 0 & 0 & 0 & 0 & 0 & 2.7 \end{pmatrix} \text{GPa} \tag{1}$$

and

$$\mathbf{C}_{\text{fcu-6}} = \begin{pmatrix} 22.08 & 6.72 & 6.99 & 0.06 & 0.09 & -0.37 \\ 6.72 & 22.08 & 6.57 & 0.14 & -0.18 & -0.70 \\ 6.99 & 6.57 & 21.88 & 0.53 & 0.32 & -0.12 \\ 0.06 & 0.14 & 0.53 & 10.03 & -0.08 & 0.05 \\ 0.09 & -0.18 & 0.32 & -0.08 & 10.38 & 0.05 \\ -0.37 & -0.70 & -0.12 & 0.05 & 0.05 & 10.10 \end{pmatrix} \text{GPa} \tag{2}$$

Note that, by design, $C_{12}$ is identically zero for the **pcu** model. An interesting point is that the **fcu-6** tensor, although calculated for an approximant with no internal crystallographic symmetry, nonetheless takes a form that is close to that expected for a cubic material. In particular, $C_{11} \simeq C_{22} \simeq C_{33}$, $C_{44} \simeq C_{55} \simeq C_{66}$, $C_{12} \simeq C_{13} \simeq C_{23}$, and $C_{4\leq i \neq j \leq 6} \simeq 0$. This correspondence is expected to become increasingly exact with increasing approximant size, and is an example of emergent symmetry as a consequence of configurational averaging.

The elastic properties of our coarse-grained models were visualised using the ELATE program.[66] We show in Supplementary Figures 2 and 3 the orientational dependence of various elastic properties.

## Supplementary Note 4

The reticular chemistry structure resource (`rcsr.anu.edu.au`) gives that only two isotropic ordered variants of the **fcu-6** topology exist; these are denoted by the labels **crs** and **hxg**. Neither topology is realisable in TRUMOF-1 since the node connectivity is not compatible with the octahedral coordination of $OZn_4$ units. Nevertheless, we interrogated the elastic properties

of both networks using the same coarse-grained parameterisation discussed above in terms of the TRUMOF-1 approximants. The orientational dependence of the Young's moduli of **fcu-6**, **crs** and **hxg** models, obtained from ELATE[66] using the elastic matrices from their GULP outputs, are shown in Supplementary Figure 4. All three moduli are relatively isotropic, with maxima along the $\langle 111 \rangle$ directions.

## Supplementary Note 5

Since there are many different approximants to the TRUMOF-1 elastic network of a given supercell size, we determined the variance in elastic properties from approximant to approximant. Our results for a set of ten $2 \times 2 \times 2$ and $4 \times 4 \times 4$ approximants are shown in Supplementary Figure 5. Here we have used the 'soft' parameterisations, which we expected to give rise the greatest variance from configuration to configuration. Because larger approximants more closely approach the true isotropic limit, there is reduced variance amongst the $4 \times 4 \times 4$ supercells relative to the $2 \times 2 \times 2$ supercells. A consequence of the larger variance amongst the smaller supercells is that they are more likely to show elastic instabilities at lower pressures.

We note for completeness that there are only three symmetry-distinct $1 \times 1 \times 1$ approximants, all of which are less elastically stable again than the $2 \times 2 \times 2$ approximants shown here. Indeed, one of the $1 \times 1 \times 1$ approximants is unstable at all pressures in our simple parameterisation because it consists of two interpenetrating frameworks between which there are no interactions.

## Supplementary Note 6

The symmetry-adapted local distortion calculations discussed in the text were repeated for the intermediate pressure of 0.5 GPa. The displacement magnitudes at this lower pressure were significantly smaller than for 1 GPa, and accordingly they were also more uniformly distributed amongst the different symmetry-adapted modes [Supplementary Figure 6]. Nevertheless the various trends noted above are preserved in these intermediate data. Our interpretation is that the compression mechanism in TRUMOF-1 becomes increasingly spatially heterogeneous as pressure is increased.

## Supplementary Figures

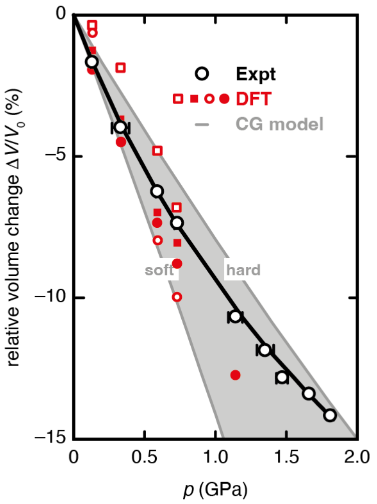


**Supplementary Figure 1:** Pressure-dependence of the relative volume change for four $2\times2\times2$ approximants (red symbols), as determined using DFT. Of these, those represented by solid circles, which extend to higher pressures, correspond to a supercell of the most stable $1 \times 1 \times 1$ approximant. The solid red squares correspond to those data reported in the main text. The experimental $V(p)$ equation of state obtained using single-crystal X-ray diffraction measurements (open black circles) and third-order Birch–Murnaghan fit (solid black line) are as shown in the main text. The errors for $p$ and $V$ in the experimental data are given for the uncertainties in the measurement as described in Methods.

**Spatial dependence of Young's modulus**

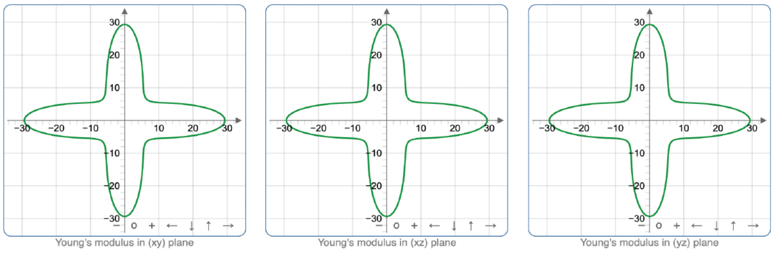


**Spatial dependence of linear compressibility**

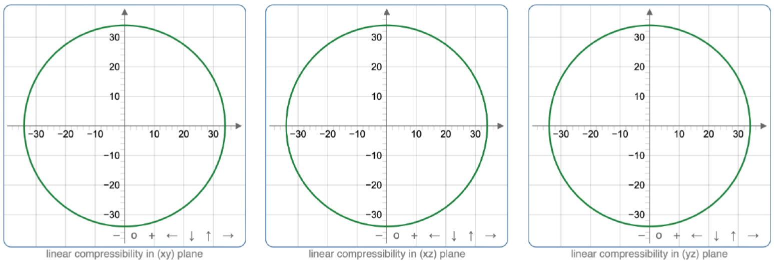


**Spatial dependence of shear modulus**

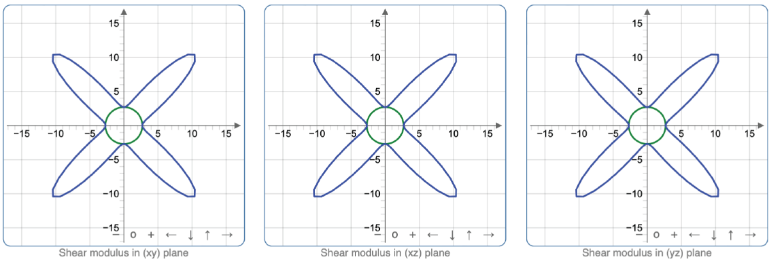


**Spatial dependence of Poisson's ratio**

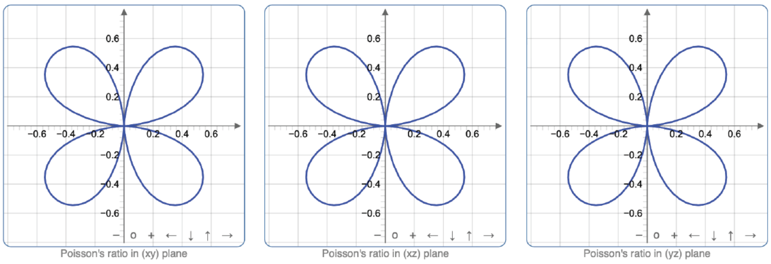


**Supplementary Figure 2:** Spatial dependencies of Young's modulus, linear compressibility, shear modulus and Poisson's ratio of **pcu** model with hard parameters at 0 GPa via ELATE

Spatial dependence of Young's modulus

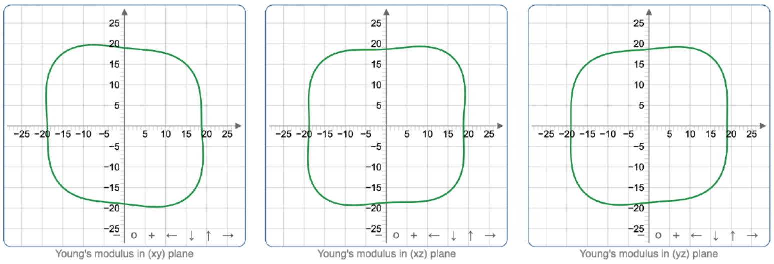


Spatial dependence of linear compressibility

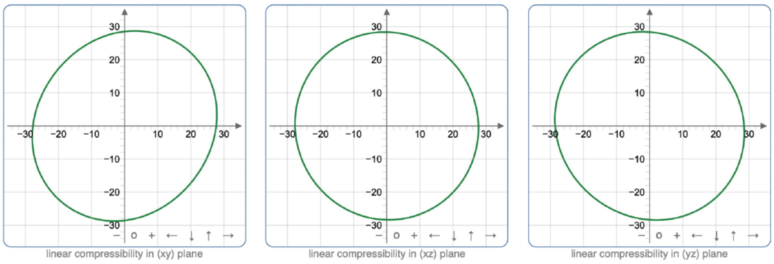


Spatial dependence of shear modulus

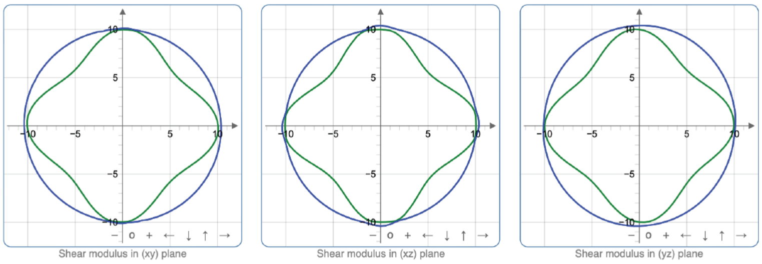


Spatial dependence of Poisson's ratio

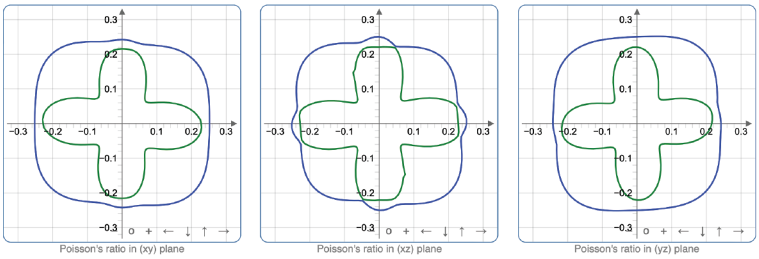


**Supplementary Figure 3:** Spatial dependencies of Young's modulus, linear compressibility, shear modulus and Poisson's ratio of **fcu-6** model with hard parameters at 0 GPa via ELATE

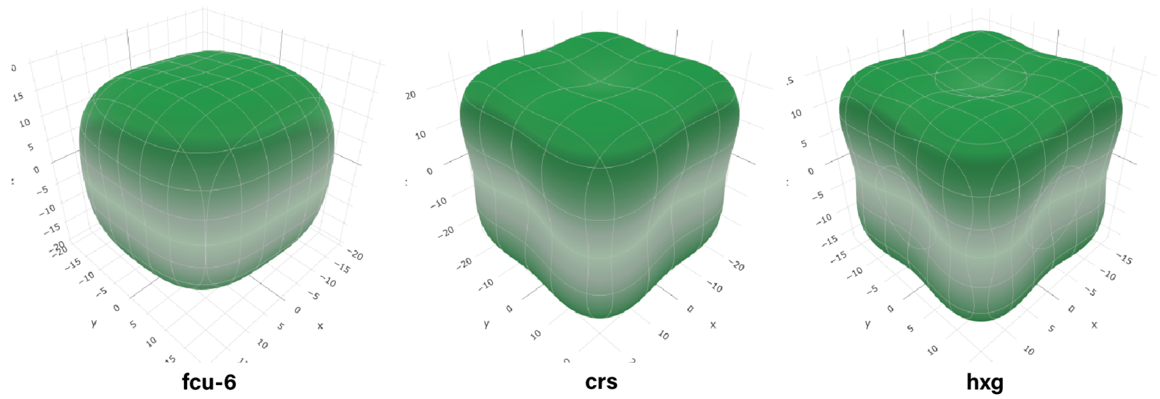


**Supplementary Figure 4:** Spatial dependencies of Young's modulus of **fcu-6**, **crs** and **hxg** models with hard parameters at 0 GPa via ELATE.

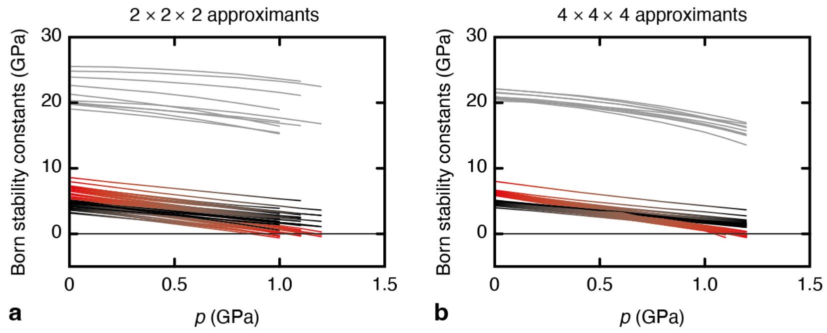


**Supplementary Figure 5:** Pressure dependence of the Born stability constants for our coarse-grained TRUMOF-1 models, calculated using the 'soft' parameterisation of our lattice dynamical model, and with values for ten $2 \times 2 \times 2$ and $4 \times 4 \times 4$ approximants overlaid in panels **a** and **b**, respectively. Formally, the absence of crystallographic symmetry in the approximant structure allows arbitrary mixing of the eigenstates of the elastic tensor. We have coloured the stability constants by similarity to the $C_{11} + 2C_{12}, C_{11} - C_{12}, C_{44}$ eigenstates, as in Fig. 3d of the main text.

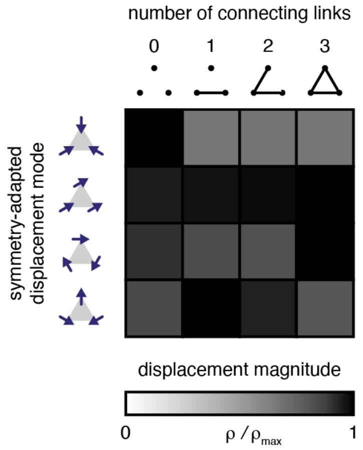


**Supplementary Figure 6:** Relative population $\rho/\rho_{\mathrm{max}}$ for each symmetry-adapted mode as a function of the local elastic connectivity; the values given here are taken as the average over the mode displacements activated on compression to 0.5 GPa amongst ten $4 \times 4 \times 4$ approximants.

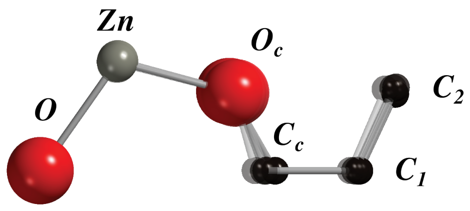


**Supplementary Figure 7:** Representations of the asymmetric unit of TRUMOF-1, overlaid with decreasing opacity for each pressure point. Atom labels are shown using the same terminology of Fig. 2 of the main text. The correspondence to atom labels applied in our crystallographic refinements is as follows: O = O1, Zn = Zn1; $O_c$ = O2; $C_c$ = C1; $C_1$ = C2; $C_2$ = C3.

## Supplementary Tables

**Supplementary Table 1:** Pressure-dependent unit-cell parameters as determined using DFT geometry optimisation under pressure for three 2×2×2 approximants and for the $2 \times 2 \times 2$ supercell of the $P\bar{4}$ ground-state structure.

| Approximant | $p$ (GPa) | $a$ (Å) | $b$ (Å) | $c$ (Å) | $\alpha$ (°) | $\beta$ (°) | $\gamma$ (°) | $V$ (Å$^3$) |
|---|---|---|---|---|---|---|---|---|
| II | 0 | 30.88 | 31.39 | 31.43 | 90.44 | 89.20 | 89.89 | 30458 |
| | 0.13 | 30.81 | 31.32 | 31.37 | 90.44 | 89.23 | 89.87 | 30263 |
| | 0.33 | 30.31 | 30.83 | 31.12 | 90.26 | 89.78 | 89.73 | 29083 |
| | 0.59 | 29.99 | 30.31 | 30.75 | 90.09 | 90.23 | 89.67 | 27954 |
| | 0.73 | 29.85 | 29.95 | 30.47 | 89.98 | 90.54 | 89.63 | 27242 |
| V | 0 | 31.26 | 30.98 | 31.55 | 90.08 | 89.98 | 89.24 | 30558 |
| | 0.13 | 31.22 | 30.94 | 31.51 | 90.07 | 89.98 | 89.23 | 30441 |
| | 0.33 | 31.07 | 30.78 | 31.36 | 90.04 | 89.97 | 89.18 | 29988 |
| | 0.59 | 30.75 | 30.39 | 31.13 | 89.97 | 89.98 | 89.08 | 29087 |
| | 0.73 | 30.54 | 30.09 | 31.00 | 89.93 | 90.03 | 89.01 | 28478 |
| XIII | 0 | 31.73 | 30.26 | 31.06 | 89.79 | 90.47 | 90.22 | 29820 |
| | 0.13 | 31.63 | 30.10 | 30.94 | 89.79 | 90.41 | 90.22 | 29448 |
| | 0.33 | 31.45 | 29.76 | 30.69 | 89.88 | 90.38 | 90.23 | 28722 |
| | 0.59 | 31.29 | 29.21 | 30.35 | 89.98 | 90.34 | 90.37 | 27736 |
| | 0.73 | 31.22 | 28.98 | 30.22 | 89.99 | 90.32 | 90.41 | 27336 |
| $P\bar{4}$ | 0 | 29.30 | 29.29 | 30.26 | 90.00 | 90.00 | 90.00 | 25967 |
| | 0.13 | 29.10 | 29.10 | 30.08 | 90.00 | 90.00 | 90.00 | 25465 |
| | 0.33 | 28.82 | 28.82 | 29.85 | 90.00 | 90.00 | 90.00 | 24802 |
| | 0.59 | 28.52 | 28.52 | 29.57 | 90.00 | 90.00 | 90.00 | 24057 |
| | 0.73 | 28.38 | 28.38 | 29.40 | 90.00 | 90.00 | 90.00 | 23687 |
| | 1.14 | 28.07 | 28.07 | 28.77 | 90.00 | 90.00 | 90.00 | 22658 |

**Supplementary Table 2:** TRUMOF-1 pore volume and contents as a function of pressure. The subscripts '1' and '2' correspond to large and small pores, respectively.

| $p$ (GPa) | $V_1$(Å$^3$) | $n_1$(e$^-$) | $V_2$(Å$^3$) | $n_2$(e$^-$) | $n$(DMF) / unit cell |
|---|---|---|---|---|---|
| 0.13(1) | 844 | 115 | 104 | 40 | 4 |
| 0.33(6) | 773 | 84 | 112 | 40 | 3 |
| 0.59(1) | 721 | 82 | 100 | 40 | 3 |
| 0.73(2) | 705 | 115 | 88 | 28 | 4 |
| 1.14(5) | 528 | 64 | 100 | 44 | 2.7 |
| 1.35(6) | 448 | 128 | – | – | 3 |
| 1.47(4) | – | – | – | – | – |
| 1.66(1) | 432 | 180 | – | – | 2 |
| 1.81(2) | 420 | 144 | 148 | 40 | 4.5 |

**Supplementary Table 3:** Symmetry-adapted mode displacement magnitudes of neighbouring node triplets in $4 \times 4 \times 4$ approximants of TRUMOF-1 on compression to 1 GPa. For each cell, the absolute displacement amplitude is given in units of Å$^2$, and the relative amplitude $\rho/\rho_{\max}$ is indicated in italics underneath.

| number of elastic links | 0 | 1 | 2 | 3 |
|---|---|---|---|---|
| breathing ($A_1'$) | 0.66(6) | 0.164(14) | 0.095(8) | 0.099(9) |
| | *100(8)%* | *25(2)%* | *14.4(12)%* | *14.8(13)%* |
| translations ($E'$) | 0.66(5) | 0.80(5) | 0.95(6) | 1.09(8) |
| | *61(5)%* | *74(5)%* | *87(6)%* | *100(7)%* |
| rotations ($A_2'$) | 0.24(2) | 0.27(2) | 0.25(2) | 0.32(3) |
| | *75(7)%* | *84(7)%* | *77(6)%* | *100(8)%* |
| bending ($E'$) | 0.62(4) | 0.55(3) | 0.268(14) | 0.047(4) |
| | *100(7)%* | *88(5)%* | *43(2)%* | *7.6(6)%* |

**Supplementary Table 4:** Crystal data and structure refinement details for TRUMOF-1 at 0.13(1) GPa.

| | |
|---|---|
| Empirical formula | $C_{24}$ $H_9$ $O_{13}$ $Zn_4$ |
| Formula weight / g mol$^{-1}$ | 766.79 |
| Temperature / K | 293(2) |
| Crystal system | cubic |
| Space group | $F\bar{4}3m$ |
| *a* / Å | 15.4042(4) |
| $\alpha$ / ° | 90 |
| $V$ / Å$^3$ | 3655.3(3) |
| Z | 4 |
| $\rho_{calc}$ / g cm$^{-3}$ | 1.393 |
| $\mu$ / mm$^{-1}$ | 0.921 |
| F(000) | 1508.0 |
| Crystal size / mm$^3$ | 0.16 × 0.125 × 0.05 |
| Radiation | synchrotron ($\lambda$ = 0.4859 Å) |
| 2$\theta$ range for data collection / ° | 3.13 – 35.37 |
| Index ranges | $-19 \leq h \leq 19, -12 \leq k \leq 12, -19 \leq l \leq 19$ |
| Reflections collected | 3384 |
| Independent reflections | 426 [$R_{int}$ = 0.01097, $R_\sigma$ = 0.0598] |
| Data/restraints/parameters | 426/12/29 |
| Goodness-of-fit on $F^2$ | 1.174 |
| Final $R$ indexes [$I > 2\sigma(I)$] | $R_1$ = 0.0517, $wR_2$ = 0.1858 |
| Final $R$ indexes [all data] | $R_1$ = 0.0586, $wR_2$ = 0.1936 |
| Largest diff. peak/hole / e Å$^{-3}$ | 0.48/−0.19 |
| Flack parameter | 0.0(3) |

**Supplementary Table 5:** Crystal data and structure refinement details for TRUMOF-1 at 0.33(6) GPa.

| | |
|---|---|
| Empirical formula | $C_{24}$ $H_9$ $O_{13}$ $Zn_4$ |
| Formula weight / g mol$^{-1}$ | 766.79 |
| Temperature / K | 293(2) |
| Crystal system | cubic |
| Space group | $F\bar{4}3m$ |
| $a$ / Å | 15.2819(6) |
| $\alpha$ / ° | 90 |
| $V$ / Å$^3$ | 3568.6(4) |
| $Z$ | 4 |
| $\rho_{calc}$ / g cm$^{-3}$ | 1.427 |
| $\mu$ / mm$^{-1}$ | 0.943 |
| F(000) | 1508.0 |
| Crystal size / mm$^3$ | 0.16 × 0.125 × 0.055 |
| Radiation | Synchrotron ($\lambda$ = 0.4859 Å) |
| $2\theta$ range for data collection / ° | 3.156 – 35.314 |
| Index ranges | $-19 \leq h \leq 19, -12 \leq k \leq 12, -19 \leq l \leq 19$ |
| Reflections collected | 2695 |
| Independent reflections | 419 [$R_{int}$ = 0.1105, $R_\sigma$ = 0.0918] |
| Data/restraints/parameters | 419/6/17 |
| Goodness-of-fit on $F^2$ | 0.991 |
| Final $R$ indexes [$I > 2\sigma(I)$] | $R_1$ = 0.0668, $wR_2$ = 0.1719 |
| Final $R$ indexes [all data] | $R_1$ = 0.0844, $wR_2$ = 0.1907 |
| Largest diff. peak/hole / e Å$^{-3}$ | 0.47/−0.37 |
| Flack parameter | −0.1(4) |

**Supplementary Table 6:** Crystal data and structure refinement details for TRUMOF-1 at 0.59(1) GPa.

| | |
|---|---|
| Empirical formula | $C_{24}$ $H_9$ $O_{13}$ $Zn_4$ |
| Formula weight / g mol$^{-1}$ | 766.79 |
| Temperature / K | 293(2) |
| Crystal system | cubic |
| Space group | $F\bar{4}3m$ |
| *a* / Å | 15.1599(6) |
| $\alpha$ / ° | 90 |
| $V$ / Å$^3$ | 3484.1(4) |
| Z | 4 |
| $\rho_{calc}$ / g cm$^{-3}$ | 1.462 |
| $\mu$ / mm$^{-1}$ | 0.966 |
| F(000) | 1508.0 |
| Crystal size / mm$^3$ | 0.16 × 0.125 × 0.05 |
| Radiation | Synchrotron ($\lambda$ = 0.4859 Å) |
| $2\theta$ range for data collection / ° | 3.182 – 35.2 |
| Index ranges | $-18 \leq h \leq 18, -12 \leq k \leq 12, -18 \leq l \leq 18$ |
| Reflections collected | 2627 |
| Independent reflections | 404 [$R_{int}$ = 0.1646, $R_\sigma$ = 0.0953] |
| Data/restraints/parameters | 404/6/29 |
| Goodness-of-fit on $F^2$ | 0.976 |
| Final $R$ indexes [$I > 2\sigma(I)$] | $R_1$ = 0.0864, $wR_2$ = 0.2197 |
| Final $R$ indexes [all data] | $R_1$ = 0.1153, $wR_2$ = 0.2554 |
| Largest diff. peak/hole / e Å$^{-3}$ | 0.48/−0.17 |
| Flack parameter | −0.3(7) |

**Supplementary Table 7:** Crystal data and structure refinement details for TRUMOF-1 at 0.73(2) GPa.

| | |
|---|---|
| Empirical formula | $C_{24}$ $H_9$ $O_{13}$ $Zn_4$ |
| Formula weight / g $mol^{-1}$ | 766.79 |
| Temperature / K | 293(2) |
| Crystal system | cubic |
| Space group | $F\bar{4}3m$ |
| *a* / Å | 15.0989(6) |
| $\alpha$ / ° | 90 |
| $V$ / Å$^3$ | 3442.2(4) |
| Z | 4 |
| $\rho_{calc}$ / g $cm^{-3}$ | 1.480 |
| $\mu$ / $mm^{-1}$ | 0.978 |
| F(000) | 1508.0 |
| Crystal size / $mm^3$ | 0.16 $\times$ 0.125 $\times$ 0.05 |
| Radiation | Synchrotron ($\lambda$ = 0.4859 Å) |
| $2\theta$ range for data collection / ° | 3.194 – 35.348 |
| Index ranges | $-18 \leq h \leq 18, -12 \leq k \leq 12, -18 \leq l \leq 18$ |
| Reflections collected | 3296 |
| Independent reflections | 405 [$R_{int}$ = 0.2261, $R_\sigma$ = 0.2165] |
| Data/restraints/parameters | 405/6/17 |
| Goodness-of-fit on $F^2$ | 0.862 |
| Final $R$ indexes [$I > 2\sigma(I)$] | $R_1$ = 0.0926, $wR_2$ = 0.1806 |
| Final $R$ indexes [all data] | $R_1$ = 0.1246, $wR_2$ = 0.2068 |
| Largest diff. peak/hole / e Å$^{-3}$ | 0.50/$-$0.19 |
| Flack parameter | 0.3(5) |

**Supplementary Table 8:** Crystal data and structure refinement details for TRUMOF-1 at 1.14(5) GPa.

| | |
|---|---|
| Empirical formula | $C_{24}$ $H_9$ $O_{13}$ $Zn_4$ |
| Formula weight / g mol$^{-1}$ | 766.79 |
| Temperature / K | 293(2) |
| Crystal system | cubic |
| Space group | $F\bar{4}3m$ |
| *a* / Å | 14.9164(10) |
| $\alpha$ / ° | 90 |
| $V$ / Å$^3$ | 3318.9(7) |
| Z | 4 |
| $\rho_{calc}$ / g cm$^{-3}$ | 1.535 |
| $\mu$ / mm$^{-1}$ | 1.014 |
| F(000) | 1508.0 |
| Crystal size / mm$^3$ | 0.16 × 0.125 × 0.05 |
| Radiation | Synchrotron ($\lambda$ = 0.4859 Å) |
| $2\theta$ range for data collection / ° | 3.234 – 35.324 |
| Index ranges | $-18 \leq h \leq 18, -18 \leq k \leq 18, -12 \leq l \leq 11$ |
| Reflections collected | 2431 |
| Independent reflections | 391 [$R_{\text{int}}$ = 0.1754, $R_\sigma$ = 0.1013] |
| Data/restraints/parameters | 391/6/17 |
| Goodness-of-fit on $F^2$ | 0.860 |
| Final $R$ indexes [$I > 2\sigma(I)$] | $R_1$ = 0.0901, $wR_2$ = 0.2562 |
| Final $R$ indexes [all data] | $R_1$ = 0.1369, $wR_2$ = 0.3237 |
| Largest diff. peak/hole / e Å$^{-3}$ | 0.68/$-$0.28 |
| Flack parameter | 0.6(7) |

**Supplementary Table 9:** Crystal data and structure refinement details for TRUMOF-1 at 1.35(6) GPa.

| | |
|---|---|
| Empirical formula | $C_{24}$ $H_9$ $O_{13}$ $Zn_4$ |
| Formula weight / g mol$^{-1}$ | 766.79 |
| Temperature / K | 293(2) |
| Crystal system | cubic |
| Space group | $F\bar{4}3m$ |
| *a* / Å | 14.8504(12) |
| $\alpha$ / ° | 90 |
| $V$ / Å$^3$ | 3275.0(8) |
| Z | 4 |
| $\rho_{calc}$ / g cm$^{-3}$ | 1.555 |
| $\mu$ / mm$^{-1}$ | 1.028 |
| F(000) | 1508.0 |
| Crystal size / mm$^3$ | 0.16 × 0.125 × 0.05 |
| Radiation | Synchrotron ($\lambda$ = 0.4859 Å) |
| $2\theta$ range for data collection / ° | 3.248 – 28.022 |
| Index ranges | $-14 \leq h \leq 14, -10 \leq k \leq 10, -14 \leq l \leq 14$ |
| Reflections collected | 1618 |
| Independent reflections | 214 [$R_{\text{int}}$ = 0.5834, $R_\sigma$ = 0.4449] |
| Data/restraints/parameters | 214/7/17 |
| Goodness-of-fit on $F^2$ | 1.016 |
| Final $R$ indexes [$I > 2\sigma(I)$] | $R_1$ = 0.1726, $wR_2$ = 0.2684 |
| Final $R$ indexes [all data] | $R_1$ = 0.1882, $wR_2$ = 0.3179 |
| Largest diff. peak/hole / e Å$^{-3}$ | 0.75/$-$0.24 |
| Flack parameter | $-$1.3(10) |

**Supplementary Table 10:** Crystal data and structure refinement details for TRUMOF-1 at 1.47(4) GPa.

| | |
|---|---|
| Empirical formula | $C_{24}$ $H_9$ $O_{13}$ $Zn_4$ |
| Formula weight / g mol$^{-1}$ | 766.79 |
| Temperature / K | 293(2) |
| Crystal system | cubic |
| Space group | $F\bar{4}3m$ |
| *a* / Å | 14.7948(14) |
| $\alpha$ / ° | 90 |
| $V$ / Å$^3$ | 3238.3(9) |
| Z | 4 |
| $\rho_{calc}$ / g cm$^{-3}$ | 1.573 |
| $\mu$ / mm$^{-1}$ | 1.040 |
| F(000) | 1508.0 |
| Crystal size / mm$^3$ | 0.16 × 0.125 × 0.05 |
| Radiation | Synchrotron ($\lambda$ = 0.4859 Å) |
| $2\theta$ range for data collection / ° | 3.26 – 27.932 |
| Index ranges | $-14 \leq h \leq 14, -14 \leq k \leq 14, -10 \leq l \leq 10$ |
| Reflections collected | 1310 |
| Independent reflections | 208 [$R_{\text{int}}$ = 0.4486, $R_\sigma$ = 0.3995] |
| Data/restraints/parameters | 208/7/17 |
| Goodness-of-fit on $F^2$ | 1.029 |
| Final $R$ indexes [$I > 2\sigma(I)$] | $R_1$ = 0.1614, $wR_2$ = 0.3796 |
| Final $R$ indexes [all data] | $R_1$ = 0.1751, $wR_2$ = 0.4208 |
| Largest diff. peak/hole / e Å$^{-3}$ | 0.40/$-$0.27 |
| Flack parameter | $-$1(2) |

**Supplementary Table 11:** Crystal data and structure refinement details for TRUMOF-1 at 1.66(1) GPa.

| | |
|---|---|
| Empirical formula | $C_{24}$ $H_9$ $O_{13}$ $Zn_4$ |
| Formula weight / g mol$^{-1}$ | 766.79 |
| Temperature / K | 293(2) |
| Crystal system | cubic |
| Space group | $F\bar{4}3m$ |
| *a* / Å | 14.7629(14) |
| $\alpha$ / ° | 90 |
| $V$ / Å$^3$ | 3217.5(9) |
| Z | 4 |
| $\rho_{calc}$ / g cm$^{-3}$ | 1.583 |
| $\mu$ / mm$^{-1}$ | 1.046 |
| F(000) | 1508.0 |
| Crystal size / mm$^3$ | 0.16 $\times$ 0.125 $\times$ 0.05 |
| Radiation | Synchrotron ($\lambda$ = 0.4859 Å) |
| 2$\theta$ range for data collection / ° | 3.266 – 21.46 |
| Index ranges | $-8 \leq h \leq 8, -11 \leq k \leq 11, -11 \leq l \leq 11$ |
| Reflections collected | 643 |
| Independent reflections | 101[$R_{int}$=0.4891, $R_\sigma$=0.3422] |
| Data/restraints/parameters | 101/7/17 |
| Goodness-of-fit on $F^2$ | 1.261 |
| Final $R$ indexes [$I > 2\sigma(I)$] | $R_1$ = 0.1663, $wR_2$ = 0.3257 |
| Final $R$ indexes [all data] | $R_1$ = 0.1676, $wR_2$ = 0.3476 |
| Largest diff. peak/hole / e Å$^{-3}$ | 0.66/–0.54 |
| Flack parameter | 1.5(18) |

**Supplementary Table 12:** Crystal data and structure refinement details for TRUMOF-1 at 1.81(2) GPa.

| | |
|---|---|
| Empirical formula | $C_{24}$ $H_9$ $O_{13}$ $Zn_4$ |
| Formula weight / g mol$^{-1}$ | 766.79 |
| Temperature / K | 293(2) |
| Crystal system | cubic |
| Space group | $F\bar{4}3m$ |
| *a* / Å | 14.7185(16) |
| $\alpha$ / ° | 90 |
| $V$ / Å$^3$ | 3188.5(10) |
| Z | 4 |
| $\rho_{calc}$ / g cm$^{-3}$ | 1.597 |
| $\mu$ / mm$^{-1}$ | 1.056 |
| F(000) | 1508.0 |
| Crystal size / mm$^3$ | 0.16 $\times$ 0.125 $\times$ 0.05 |
| Radiation | Synchrotron ($\lambda$ = 0.4859 Å) |
| $2\theta$ range for data collection / ° | 3.276 – 21.526 |
| Index ranges | $-11 \leq h \leq 11, -7 \leq k \leq 7, -11 \leq l \leq 11$ |
| Reflections collected | 640 |
| Independent reflections | 101[$R_{int}$=0.2467, $R_\sigma$=0.2442] |
| Data/restraints/parameters | 101/7/17 |
| Goodness-of-fit on $F^2$ | 1.613 |
| Final $R$ indexes [$I > 2\sigma(I)$] | $R_1$ = 0.1204, $wR_2$ = 0.3643 |
| Final $R$ indexes [all data] | $R_1$ = 0.1244, $wR_2$ = 0.3800 |
| Largest diff. peak/hole / e Å$^{-3}$ | 0.66/–0.23 |
| Flack parameter | 1(2) |

**Supplementary Table 13:** TRUMOF-1 experimental pressure $p$ and third order Birch-Murnaghan fit pressure $p_{\text{3rd}}$ as functions of unit-cell volume $V$.

| $V$(Å$^3$) | $p$ (GPa) | $p_{\text{3rd}}$ (GPa) |
|---|---|---|
| 3655.3(3) | 0.13(1) | 0.131 |
| 3568.6(4) | 0.33(6) | 0.3437 |
| 3484.1(4) | 0.59(1) | 0.5879 |
| 3442.2(4) | 0.73(2) | 0.7232 |
| 3318.9(7) | 1.14(5) | 1.1871 |
| 3275.0(8) | 1.35(6) | 1.3791 |
| 3238.3(9) | 1.47(4) | 1.5518 |
| 3217.5(9) | 1.66(1) | 1.6556 |
| 3188.5(10) | 1.81(2) | 1.8061 |

## Supplementary References


63. Perdew, J. P. *et al.* Restoring the density-gradient expansion for exchange in solids and surfaces. *Phys. Rev. Lett.* **100**, 136406 (2008).

64. van der Sluis, P. & Spek, A. L. BYPASS: an effective method for the refinement of crystal structures containing disordered solvent regions. *Acta Cryst. A* **46**, 194–201 (1990).

65. Palmer, D. Crystalmaker. *CrystalMaker Software Ltd, Begbroke, Oxfordshire, England* (2014).

66. Gaillac, R., Pullumbi, P. & Coudert, F.-X. ELATE: an open-source online application for analysis and visualization of elastic tensors. *J. Phys.: Cond. Matt.* **28**, 275201 (2016).
