# Supplementary material for: Enhanced elastic stability of a topologically disordered crystalline metal–organic framework
Source: Nat Mater. 2024 Jul 23;23(9):1245–51. doi: 10.1038/s41563-024-01960-7 (PMC11364505; doi:10.1038/s41563-024-01960-7)

checkCIF/PLATON report

Structure factors have been supplied for datablock(s) trumof1_03_0

THIS REPORT IS FOR GUIDANCE ONLY. IF USED AS PART OF A REVIEW PROCEDURE FOR PUBLICATION, IT SHOULD NOT REPLACE THE EXPERTISE OF AN EXPERIENCED CRYSTALLOGRAPHIC REFEREE.

No syntax errors found. CIF dictionary Interpreting this report

Datablock: trumof1_03_0

Bond precision:	Zn- O = 0.0012 A		Wavelength=0.48590
Cell:	a=15.4042(4)	b=15.4042(4)	c=15.4042(4)
	alpha=90	beta=90	gamma=90
Temperature:	293 K		

	Calculated	Reported
Volume	3655.3(3)	3655.3(3)
Space group	F -4 3 m	F -4 3 m
Hall group	F -4 2 3	F -4 2 3
Moiety formula	C6 O13 Zn4, 3(C6 H3) [+ solvent]	C24 H9 O13 Zn4, [+ solventS]
Sum formula	C24 H9 O13 Zn4 [+ solvent]	C24 H9 O13 Zn4
Mr	766.87	766.79
Dx,g cm-3	1.393	1.393
Z	4	4
Mu (mm-1)	0.933	0.921
F000	1508.0	1508.0
F000'	1512.81	
h,k,lmax	19,19,19	19,12,19
Nref	420[232]	426
Tmin,Tmax	0.871,0.955	0.306,1.000
Tmin'	0.863	

Correction method= # Reported T Limits: Tmin=0.306 Tmax=1.000
AbsCorr = MULTI-SCAN

Data completeness= 1.84/1.01 Theta(max)= 17.685

R(reflections)= 0.0517(320) wR2(reflections)= 0.1936(426)

S = 1.090 Npar= 17

The following ALERTS were generated. Each ALERT has the format
test-name_ALERT_alert-type_alert-level.
Click on the hyperlinks for more details of the test.

Alert level A

```
ATOM007_ALERT_1_A  _atom_site_aniso_label is missing
            Unique label identifying the atom site.
```

Author Response: All atoms are refined isotropically due to loss of information/lack of completeness arising from the DAC set-up.

Alert level B

```
PLAT201_ALERT_2_B Isotropic non-H Atoms in Main Residue(s) .......          2 Report
              Zn1       O1
```

Author Response: Due to the limitations of high-pressure diffraction experiments, the calculation of anisotropic displacement parameters becomes less accurate. Therefore all atoms were modelled isotropically.

Alert level C

```
PLAT094_ALERT_2_C Ratio of Maximum / Minimum Residual Density ....       2.54 Report
PLAT202_ALERT_3_C Isotropic non-H Atoms in Anion/Solvent .........          2 Check
              C2      C3
PLAT242_ALERT_2_C Low    'MainMol' Ueq as Compared to Neighbors of        Zn1 Check
```

Alert level G

```
ABSMU01_ALERT_1_G  Calculation of _exptl_absorpt_correction_mu
                not performed for this radiation type.
PLAT003_ALERT_2_G Number of Uiso or Uij Restrained non-H Atoms ...          2 Report
PLAT032_ALERT_4_G Std. Uncertainty on Flack Parameter Value High .      0.300 Report
PLAT042_ALERT_1_G Calc. and Reported MoietyFormula Strings  Differ     Please Check
PLAT072_ALERT_2_G SHELXL First  Parameter in WGHT  Unusually Large       0.12 Report
PLAT092_ALERT_4_G Check: Wavelength Given is not Cu,Ga,Mo,Ag,In Ka    0.48590 Ang.
PLAT171_ALERT_4_G The CIF-Embedded .res File Contains EADP Records          1 Report
PLAT174_ALERT_4_G The CIF-Embedded .res File Contains FLAT Records          1 Report
PLAT178_ALERT_4_G The CIF-Embedded .res File Contains SIMU Records          1 Report
PLAT199_ALERT_1_G Reported _cell_measurement_temperature ..... (K)        293 Check
PLAT200_ALERT_1_G Reported   _diffrn_ambient_temperature ..... (K)        293 Check
PLAT300_ALERT_4_G Atom Site Occupancy of O2         Constrained at        0.5 Check
PLAT300_ALERT_4_G Atom Site Occupancy of C1         Constrained at        0.5 Check
PLAT300_ALERT_4_G Atom Site Occupancy of C2         Constrained at       0.75 Check
PLAT300_ALERT_4_G Atom Site Occupancy of C3         Constrained at       0.75 Check
PLAT300_ALERT_4_G Atom Site Occupancy of H3         Constrained at       0.75 Check
PLAT301_ALERT_3_G Main Residue  Disorder .............(Resd  1  )        83% Note
PLAT302_ALERT_4_G Anion/Solvent/Minor-Residue Disorder (Resd  2  )       100% Note
PLAT304_ALERT_4_G Non-Integer Number of Atoms in ..... (Resd  2  )       6.75 Check
```

```
PLAT432_ALERT_2_G Short Inter X...Y Contact  O2       ..C2       .       2.44 Ang.
                                                 1-y,1-z,x  =     12_665 Check
PLAT432_ALERT_2_G Short Inter X...Y Contact  O2       ..C3       .       2.81 Ang.
                                                 x,1-z,1-y  =     20_566 Check
PLAT432_ALERT_2_G Short Inter X...Y Contact  C1       ..C2       .       1.55 Ang.
                                                 1-y,1-z,x  =     12_665 Check
PLAT432_ALERT_2_G Short Inter X...Y Contact  C1       ..C3       .       2.54 Ang.
                                                 x,1-z,1-y  =     20_566 Check
PLAT432_ALERT_2_G Short Inter X...Y Contact  C1       ..C3       .       2.54 Ang.
                                                 1-y,1-z,x  =     12_665 Check
PLAT605_ALERT_4_G Largest Solvent Accessible VOID in the Structure        137 A**3
PLAT811_ALERT_5_G No ADDSYM Analysis: Too Many Excluded Atoms ....          ! Info
PLAT850_ALERT_4_G Check Flack Parameter Exact Value 0.00 with s.u.       0.30 Check
PLAT860_ALERT_3_G Number of Least-Squares Restraints ............           7 Note
PLAT868_ALERT_4_G ALERTS Due to the Use of _smtbx_masks Suppressed          ! Info
PLAT951_ALERT_5_G Calculated (ThMax) and CIF-Reported Kmax Differ           7 Units
PLAT984_ALERT_1_G The  O-f'=    0.0006 Deviates from the B&C-Value     0.0036 Check
PLAT984_ALERT_1_G The Zn-f'=    0.2372 Deviates from the B&C-Value     0.3042 Check
PLAT996_ALERT_1_G Non-Standard SHELXL LIST 4 Style FCF Supplied ..          ! Check
```

---

   1 **ALERT level A** = Most likely a serious problem - resolve or explain
   1 **ALERT level B** = A potentially serious problem, consider carefully
   3 **ALERT level C** = Check. Ensure it is not caused by an omission or oversight
  33 **ALERT level G** = General information/check it is not something unexpected

```
   8 ALERT type 1 CIF construction/syntax error, inconsistent or missing data
  10 ALERT type 2 Indicator that the structure model may be wrong or deficient
   3 ALERT type 3 Indicator that the structure quality may be low
  15 ALERT type 4 Improvement, methodology, query or suggestion
   2 ALERT type 5 Informative message, check
```

It is advisable to attempt to resolve as many as possible of the alerts in all categories. Often the minor alerts point to easily fixed oversights, errors and omissions in your CIF or refinement strategy, so attention to these fine details can be worthwhile. In order to resolve some of the more serious problems it may be necessary to carry out additional measurements or structure refinements. However, the purpose of your study may justify the reported deviations and the more serious of these should normally be commented upon in the discussion or experimental section of a paper or in the "special_details" fields of the CIF. checkCIF was carefully designed to identify outliers and unusual parameters, but every test has its limitations and alerts that are not important in a particular case may appear. Conversely, the absence of alerts does not guarantee there are no aspects of the results needing attention. It is up to the individual to critically assess their own results and, if necessary, seek expert advice.

Publication of your CIF in IUCr journals

A basic structural check has been run on your CIF. These basic checks will be run on all CIFs submitted for publication in IUCr journals (*Acta Crystallographica*, *Journal of Applied Crystallography*, *Journal of Synchrotron Radiation*); however, if you intend to submit to *Acta Crystallographica Section C* or *E* or *IUCrData*, you should make sure that full publication checks are run on the final version of your CIF prior to submission.

Publication of your CIF in other journals

Please refer to the *Notes for Authors* of the relevant journal for any special instructions relating to CIF submission.

PLATON version of 10/05/2023; check.def file version of 10/05/2023

Datablock trumof1_03_0 - ellipsoid plot

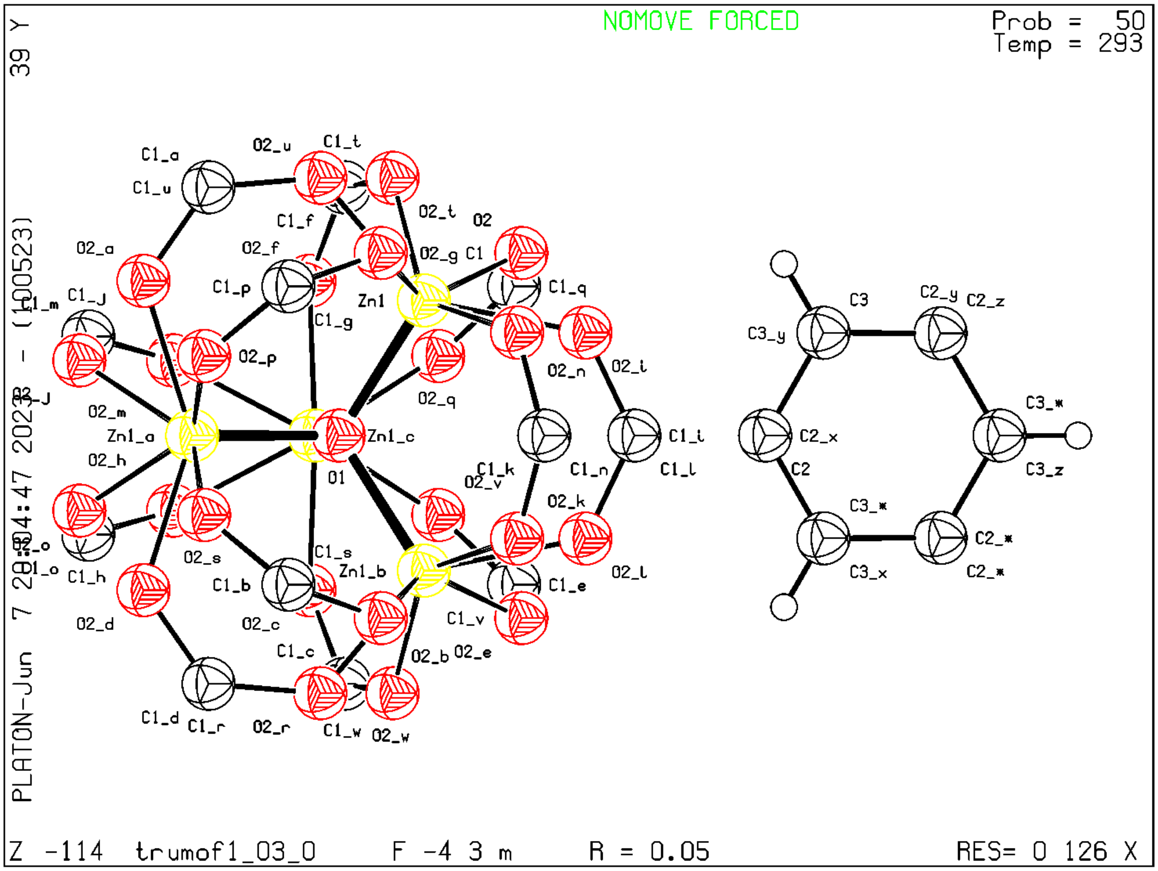

Supplement: Supplementary file 2 — Crystallographic data. [file TRUMOF1_03_checkCIF.pdf › Supplementary Data 1/TRUMOF1_03_checkCIF.pdf]
