# Supplementary material for: Enhanced elastic stability of a topologically disordered crystalline metal–organic framework
Source: Nat Mater. 2024 Jul 23;23(9):1245–51. doi: 10.1038/s41563-024-01960-7 (PMC11364505; doi:10.1038/s41563-024-01960-7)

checkCIF/PLATON report

Structure factors have been supplied for datablock(s) trumof1_08_0

THIS REPORT IS FOR GUIDANCE ONLY. IF USED AS PART OF A REVIEW PROCEDURE FOR PUBLICATION, IT SHOULD NOT REPLACE THE EXPERTISE OF AN EXPERIENCED CRYSTALLOGRAPHIC REFEREE.

No syntax errors found. CIF dictionary Interpreting this report

Datablock: trumof1_08_0

Bond precision:	Zn- O = 0.0030 A	Wavelength=0.48590	
Cell:	a=14.9164(10)	b=14.9164(10)	c=14.9164(10)
	alpha=90	beta=90	gamma=90
Temperature:	293 K		

	Calculated	Reported
Volume	3318.9(7)	3318.9(7)
Space group	F -4 3 m	F -4 3 m
Hall group	F -4 2 3	F -4 2 3
Moiety formula	C6 O13 Zn4, 3(C6 H3) [+ solvent]	C24 H9 O13 Zn4
Sum formula	C24 H9 O13 Zn4 [+ solvent]	C24 H9 O13 Zn4
Mr	766.87	766.79
Dx,g cm-3	1.535	1.535
Z	4	4
Mu (mm-1)	1.028	1.014
F000	1508.0	1508.0
F000'	1512.81	
h,k,lmax	18,18,18	18,18,12
Nref	386[214]	391
Tmin,Tmax	0.859,0.951	0.467,2.277
Tmin'	0.850	

Correction method= # Reported T Limits: Tmin=0.467 Tmax=2.277
AbsCorr = MULTI-SCAN

Data completeness= 1.83/1.01 Theta(max)= 17.662

R(reflections)= 0.0901(128) wR2(reflections)= 0.3237(391)

S = 0.860 Npar= 17

The following ALERTS were generated. Each ALERT has the format
test-name_ALERT_alert-type_alert-level.
Click on the hyperlinks for more details of the test.

Alert level A

```
ATOM007_ALERT_1_A  _atom_site_aniso_label is missing
            Unique label identifying the atom site.
```

Author Response: All atoms are refined isotropically due to loss of information/lack of completeness arising from the DAC set-up.

Alert level B

```
PLAT026_ALERT_3_B Ratio Observed / Unique Reflections (too) Low ..        33% Check
```

Author Response: The low ratio is caused by weak data beyond sin(theta)/lambda > 0.5 (i.e. low resolution).

```
PLAT201_ALERT_2_B Isotropic non-H Atoms in Main Residue(s) .......          2 Report
              Zn1       O1
```

Author Response: Due to the limitations of high-pressure diffraction experiments, the calculation of anisotropic displacement parameters becomes less accurate. Therefore all atoms were modelled isotropically.

Alert level C

```
RINTA01_ALERT_3_C  The value of Rint is greater than 0.12
            Rint given   0.175
STRVA01_ALERT_4_C           Flack test results are ambiguous.
           From the CIF: _refine_ls_abs_structure_Flack    0.600
           From the CIF: _refine_ls_abs_structure_Flack_su    0.700
PLAT020_ALERT_3_C The Value of Rint is Greater Than 0.12 ........      0.175 Report
PLAT084_ALERT_3_C High wR2 Value (i.e. > 0.25) ..................       0.32 Report
PLAT094_ALERT_2_C Ratio of Maximum / Minimum Residual Density ....       2.43 Report
PLAT202_ALERT_3_C Isotropic non-H Atoms in Anion/Solvent ........          2 Check
              C2      C3
PLAT242_ALERT_2_C Low    'MainMol' Ueq as Compared to Neighbors of        Zn1 Check
PLAT905_ALERT_3_C Negative K value in the Analysis of Variance ...    -11.602 Report
PLAT905_ALERT_3_C Negative K value in the Analysis of Variance ...     -2.278 Report
PLAT905_ALERT_3_C Negative K value in the Analysis of Variance ...     -0.363 Report
PLAT906_ALERT_3_C Large K Value in the Analysis of Variance ......      2.167 Check
PLAT907_ALERT_2_C Flack x > 0.5, Structure Needs to be Inverted? .       0.60 Check
```

Alert level G

```
ABSMU01_ALERT_1_G  Calculation of _exptl_absorpt_correction_mu
```

```
                not performed for this radiation type.
PLAT032_ALERT_4_G Std. Uncertainty on Flack Parameter Value High .      0.700 Report
PLAT042_ALERT_1_G Calc. and Reported MoietyFormula Strings  Differ     Please Check
PLAT072_ALERT_2_G SHELXL First  Parameter in WGHT  Unusually Large       0.20 Report
PLAT092_ALERT_4_G Check: Wavelength Given is not Cu,Ga,Mo,Ag,In Ka    0.48590 Ang.
PLAT171_ALERT_4_G The CIF-Embedded .res File Contains EADP Records          1 Report
PLAT174_ALERT_4_G The CIF-Embedded .res File Contains FLAT Records          1 Report
PLAT199_ALERT_1_G Reported _cell_measurement_temperature ..... (K)        293 Check
PLAT200_ALERT_1_G Reported   _diffrn_ambient_temperature ..... (K)        293 Check
PLAT300_ALERT_4_G Atom Site Occupancy of O2        Constrained at         0.5 Check
PLAT300_ALERT_4_G Atom Site Occupancy of C1        Constrained at         0.5 Check
PLAT300_ALERT_4_G Atom Site Occupancy of C2        Constrained at        0.75 Check
PLAT300_ALERT_4_G Atom Site Occupancy of C3        Constrained at        0.75 Check
PLAT300_ALERT_4_G Atom Site Occupancy of H3        Constrained at        0.75 Check
PLAT301_ALERT_3_G Main Residue  Disorder .............(Resd  1  )         83% Note
PLAT302_ALERT_4_G Anion/Solvent/Minor-Residue Disorder (Resd  2  )       100% Note
PLAT304_ALERT_4_G Non-Integer Number of Atoms in ..... (Resd  2  )       6.75 Check
PLAT432_ALERT_2_G Short Inter X...Y Contact  O2       ..C2       .       2.26 Ang.
                                                1-y,1-z,x  =     12_665 Check
PLAT432_ALERT_2_G Short Inter X...Y Contact  O2       ..C3       .       2.69 Ang.
                                                x,1-z,1-y  =     20_566 Check
PLAT432_ALERT_2_G Short Inter X...Y Contact  C1       ..C2       .       1.44 Ang.
                                                1-y,1-z,x  =     12_665 Check
PLAT432_ALERT_2_G Short Inter X...Y Contact  C1       ..C3       .       2.44 Ang.
                                                1-y,1-z,x  =     12_665 Check
PLAT432_ALERT_2_G Short Inter X...Y Contact  C1       ..C3       .       2.44 Ang.
                                                x,1-z,1-y  =     20_566 Check
PLAT605_ALERT_4_G Largest Solvent Accessible VOID in the Structure        117 A**3
PLAT764_ALERT_4_G Overcomplete CIF Bond List Detected (Rep/Expd) .       1.25 Ratio
PLAT811_ALERT_5_G No ADDSYM Analysis: Too Many Excluded Atoms ....          ! Info
PLAT860_ALERT_3_G Number of Least-Squares Restraints ............           6 Note
PLAT868_ALERT_4_G ALERTS Due to the Use of _smtbx_masks Suppressed          ! Info
PLAT916_ALERT_2_G Hooft y and Flack x Parameter Values Differ by .       0.44 Check
PLAT952_ALERT_5_G Calculated (ThMax) and CIF-Reported Lmax Differ.          6 Units
PLAT955_ALERT_1_G Reported (CIF) and Actual (FCF) Lmax Differ by .          6 Units
PLAT984_ALERT_1_G The  O-f'=    0.0006 Deviates from the B&C-Value     0.0036 Check
PLAT984_ALERT_1_G The Zn-f'=    0.2372 Deviates from the B&C-Value     0.3042 Check
```

```
  1 ALERT level A = Most likely a serious problem - resolve or explain
  2 ALERT level B = A potentially serious problem, consider carefully
 12 ALERT level C = Check. Ensure it is not caused by an omission or oversight
 32 ALERT level G = General information/check it is not something unexpected

  8 ALERT type 1 CIF construction/syntax error, inconsistent or missing data
 11 ALERT type 2 Indicator that the structure model may be wrong or deficient
 11 ALERT type 3 Indicator that the structure quality may be low
 15 ALERT type 4 Improvement, methodology, query or suggestion
  2 ALERT type 5 Informative message, check
```

It is advisable to attempt to resolve as many as possible of the alerts in all categories. Often the minor alerts point to easily fixed oversights, errors and omissions in your CIF or refinement strategy, so attention to these fine details can be worthwhile. In order to resolve some of the more serious problems it may be necessary to carry out additional measurements or structure refinements. However, the purpose of your study may justify the reported deviations and the more serious of these should normally be commented upon in the discussion or experimental section of a paper or in the "special_details" fields of the CIF. checkCIF was carefully designed to identify outliers and unusual parameters, but every test has its limitations and alerts that are not important in a particular case may appear. Conversely, the absence of alerts does not guarantee there are no aspects of the results needing attention. It is up to the individual to critically assess their own results and, if necessary, seek expert advice.

Publication of your CIF in IUCr journals

A basic structural check has been run on your CIF. These basic checks will be run on all CIFs submitted for publication in IUCr journals (*Acta Crystallographica*, *Journal of Applied Crystallography*, *Journal of Synchrotron Radiation*); however, if you intend to submit to *Acta Crystallographica Section C* or *E* or *IUCrData*, you should make sure that full publication checks are run on the final version of your CIF prior to submission.

Publication of your CIF in other journals

Please refer to the *Notes for Authors* of the relevant journal for any special instructions relating to CIF submission.

PLATON version of 10/05/2023; check.def file version of 10/05/2023

Datablock trumof1_08_0 - ellipsoid plot

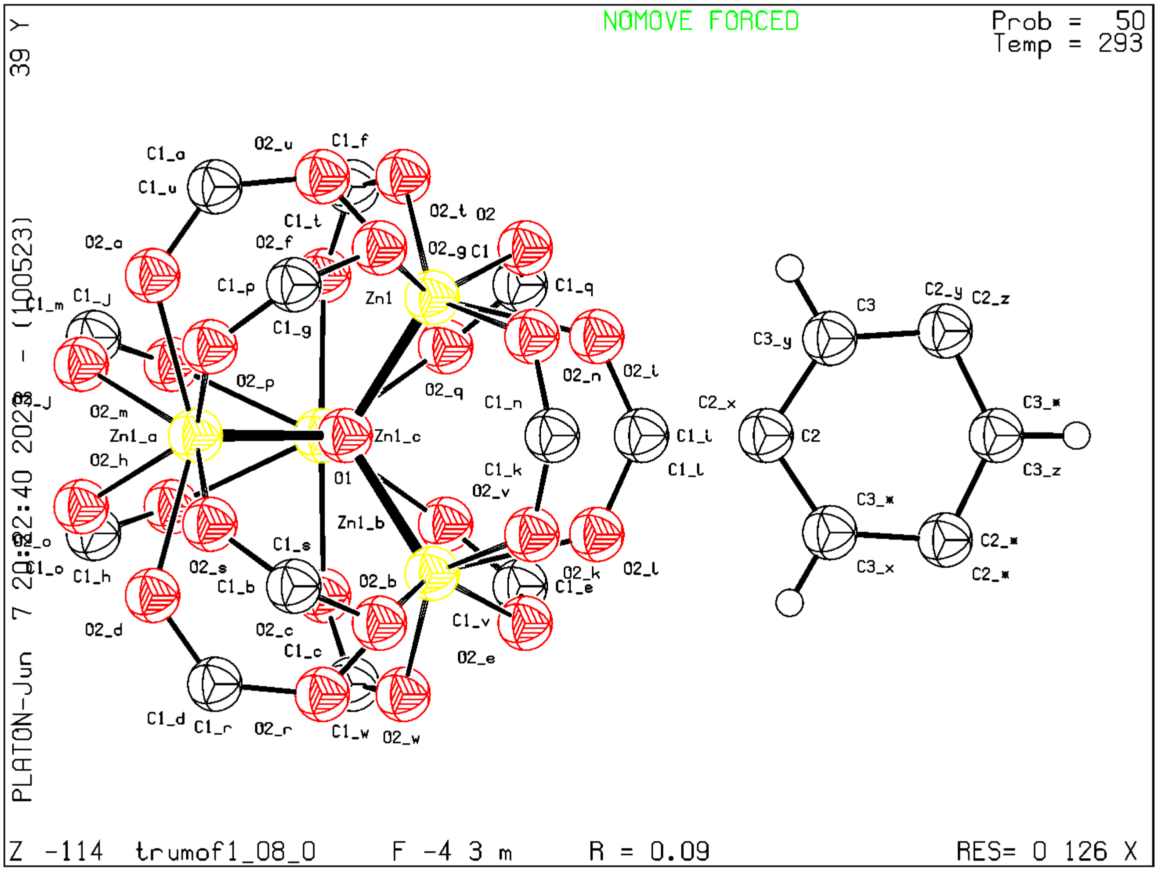

Supplement: Supplementary file 2 — Crystallographic data. [file TRUMOF1_08_checkCIF.pdf › Supplementary Data 1/TRUMOF1_08_checkCIF.pdf]
